# BUB1b impairs chemotherapy sensitivity via resistance to ferroptosis in lung adenocarcinoma

**DOI:** 10.1038/s41419-024-06914-0

**Published:** 2024-07-23

**Authors:** Yanguang Ding, Jian Gao, Jun Chen, Jinmei Ren, Jiahao Jiang, Zhiqiang Zhang, Xin Tong, Jun Zhao

**Affiliations:** 1 Department of Thoracic Surgery, The First Affiliated Hospital of Soochow University, Suzhou, China; 2 Institute of Thoracic Surgery, The First Affiliated Hospital of Soochow University, Suzhou, China; 3 Department of Thoracic Surgery, Qingpu Branch of Zhongshan Hospital, Fudan University, Shanghai, China; 4 Department of Thoracic Surgery, Zhongshan Hospital, Fudan University, Shanghai, China; 5 Department of Pharmacy, Qingpu Branch of Zhongshan Hospital, Fudan University, Shanghai, China

**Keywords:** Lung cancer, Non-small-cell lung cancer

## Abstract

BUB1 mitotic checkpoint serine/threonine kinase B (BUB1b) has been unequivocally identified as an oncogene in various cancers. However, the potential mechanism by which BUB1b orchestrates the progression of lung adenocarcinoma (LUAD) remains unclear. Here we found that both the transcript and protein levels of BUB1b were dramatically upregulated in tumor tissues and contributed to the dismal prognosis of LUAD patients. Moreover, gain- and loss-of-function assays, conducted both in vitro and in vivo, confirmed that BUB1b enhanced the viability of LUAD cells. Mechanistically, BUB1b forms a complex with OTUD3 and NRF2 and stabilizes the downstream NRF2 signaling pathway to facilitate insensitivity to ferroptosis and chemotherapy. In BALB/c nude mice bearing subcutaneous tumors that overexpress BUB1b, a combined strategy of ML385 targeting and chemotherapy achieved synergistic effects, inhibiting tumor growth and obviously improving survival. Taken together our study uncovered the underlying mechanism by which BUB1b promotes the progression of LUAD and proposed a novel strategy to enhance the efficacy of chemotherapy.

## Introduction

Lung cancer is still ranked as the major cause of cancer-related death worldwide, and lung adenocarcinoma (LUAD) is the most common histological type discovered in over 40% of all cases [1]. Apart from radical resection, such as lobectomy or segmentectomy with or without adjuvant chemotherapy or radiotherapy, more precise treatments, such as EGFR-targeted therapy and immunotherapy, have improved the survival of LUAD patients. EGFR kinase inhibitors have evolved into the third generation, osimertinib, and achieved promising results in advanced LUAD patients with EGFR mutations [2]. However, the prognosis is still dismal, with a 5-year overall survival (OS) of approximately 16% and a 5-year relapse rate ranging from approximately 20% in stage I to 50% in stage III according to the latest TNM staging system [3, 4]. Thus, a better understanding of the potential mechanism facilitating the progression of LUAD is vital for further improving prognosis.

BUB1b (BUB1 mitotic checkpoint serine/threonine kinase B) belongs to the spindle family, which is responsible for the attachment of chromosomes and mitotic spindles during mitosis [5]. It is a multifunctional protein that complexes with CDC20, BUB3, and MAD2 to abrogate the function of the anaphase-promoting complex or cyclosome [6]. Considering the critical role of BUB1b in regulating chromosome segregation, the abnormal expression of BUB1b could lead to chromosomal instability and increased cancer incidence [7]. The impact of BUB1b was also heterogeneous in different cancers. In colon adenocarcinoma, the silencing of BUB1b facilitated oncogenesis and progression, while the opposite phenomenon was observed in hepatocellular carcinoma, pancreatic ductal adenocarcinoma, and other cancers [8, 9]. While a previous study reported the oncogenic role of BUB1b in lung cancer, the underlying mechanism of BUB1b-mediated progression remains unclear.

In this study, we intended to uncover the potential mechanism by which BUB1b contributes to the progression of LUAD. The bioinformatic results and immunohistochemistry (IHC) staining of BUB1b in a tissue microarray showed that BUB1b was significantly elevated in tumor tissues in contrast to normal tissues, and LUAD patients with high BUB1b expression had a dismal prognosis. Mechanistically, BUB1b could enhance the deubiquitination of NRF2 and stabilize its protein level via the recruitment of OTUD3, which induced insensitivity to ferroptosis and conferred resistance to chemotherapy in LUAD. Moreover, the NRF2 inhibitor ML385 could achieve synergistic effects with chemotherapy in LUAD with high BUB1b expression.

## Results

### The clinical significance of BUB1b in LUAD patients

**Fig. 1:**
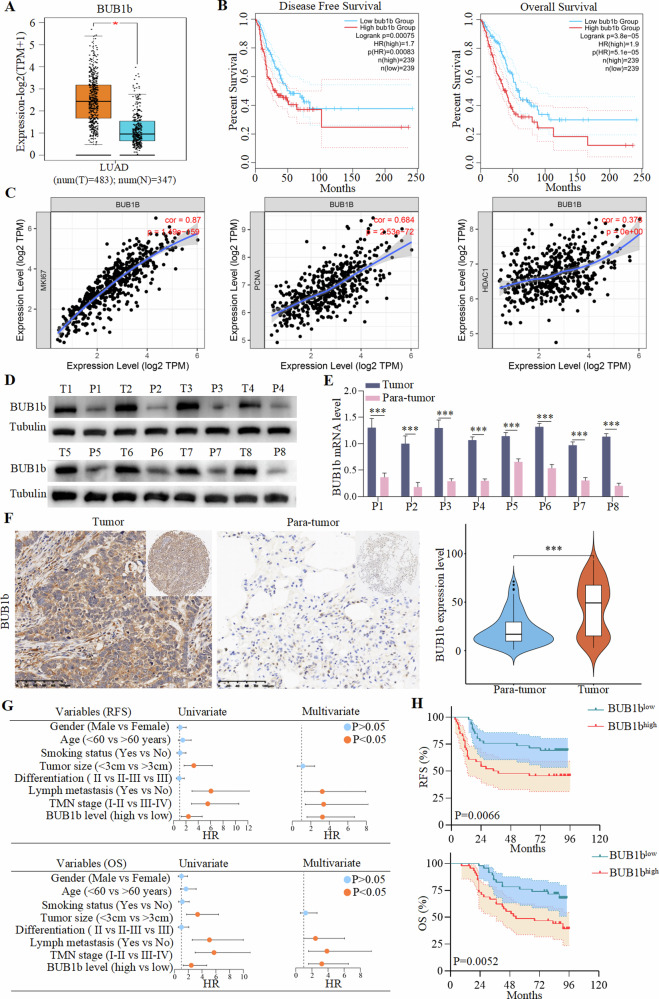
The clinical significance of BUB1b in LUAD patients. **A** Bioinformatic analysis on the expression difference of BUB1b between LUAD tumors and normal tissues based on TCGA database in GEPIA 2.0. **B** The influence of BUB1b on the prognosis of LUAD patients (DFS and OS) was analyzed based on the TCGA database in GEPIA 2.0. **C** The gene expression correlation between BUB1b and proliferation-related genes (MKI67, PCNA, and HDAC1) was analyzed using the TCGA database in TIMER 1.0. **D** Protein levels of BUB1b in eight paired tumor and para-tumor tissues of LUAD were detected by immunoblotting. **E** The mRNA level of BUB1b in eight paired tumor and para-tumor tissues of LUAD were measured by qRT-PCR. A student’s *t*-test was performed. **F** IHC staining of BUB1b in a TMA containing 92 pairs of tumor and para-tumor tissues of LUAD. The expression was calculated as the average gray value evaluated by Image-Pro Plus 6.0. **G** Multistep Cox regression analysis on the predictors for the dismal RFS and OS in this cohort of 92 LUAD patients. **H** Kaplan–Meier and log-rank analysis on the difference of prognosis between BUB1bhigh and BUB1blow groups. ***p < 0.001.

The bioinformatics analysis of the GEPIA2 database suggested that BUB1b was highly expressed in the tumor tissues of LUAD compared with normal tissues (Fig. 1A). Patients with high expression of BUB1b had shorter disease-free survival (DFS) and OS (Fig. 1B). Moreover, the relationship of BUB1b and the proliferation-related genes KI67, PCNA, and HDAC1 was explored, and it was shown that BUB1b was positively correlated with KI67 (*R* = 0.87), PCNA (*R* = 0.684), and HDAC1 (*R* = 0.375) (Fig. 1C). To verify the above transcriptome results, the expression of BUB1b was detected in eight LUAD tumor tissues and matched paratumor tissues by immunoblotting and quantitative reverse transcriptase PCR (qRT-PCR). Consistently, both the mRNA and protein levels of BUB1b were obviously elevated in tumor tissues (Fig. 1D, E). IHC staining of BUB1b was also performed in tissue samples and tissue microarray (TMA) containing 92 LUAD tumor tissues and matched para tumor tissues, and BUB1b was significantly highly expressed in tumor tissues (Fig. 1F). The clinical information of the 92 LUAD patients is presented in Supplementary Table 1. The multistep Cox regression analysis suggested an independent predictive role of BUB1b in LUAD (Fig. 1G). Additionally, the Kaplan–Meier survival analysis presented shorter recurrence-free survival (RFS) and OS in LUAD patients with high expression of BUB1b (Fig. 1H). Also, LUAD tumor tissues with high expression of BUB1b had a higher risk of lymph node metastasis and TNM III–IV stage (Supplementary Fig. 1A). Thus, the bioinformatic analysis and further verification in LUAD tissues confirmed the high expression of BUB1b, which could indicate a dismal prognosis in LUAD patients.

### BUB1b regulated the viability of LUAD cells in vitro and in vivo

**Fig. 2:**
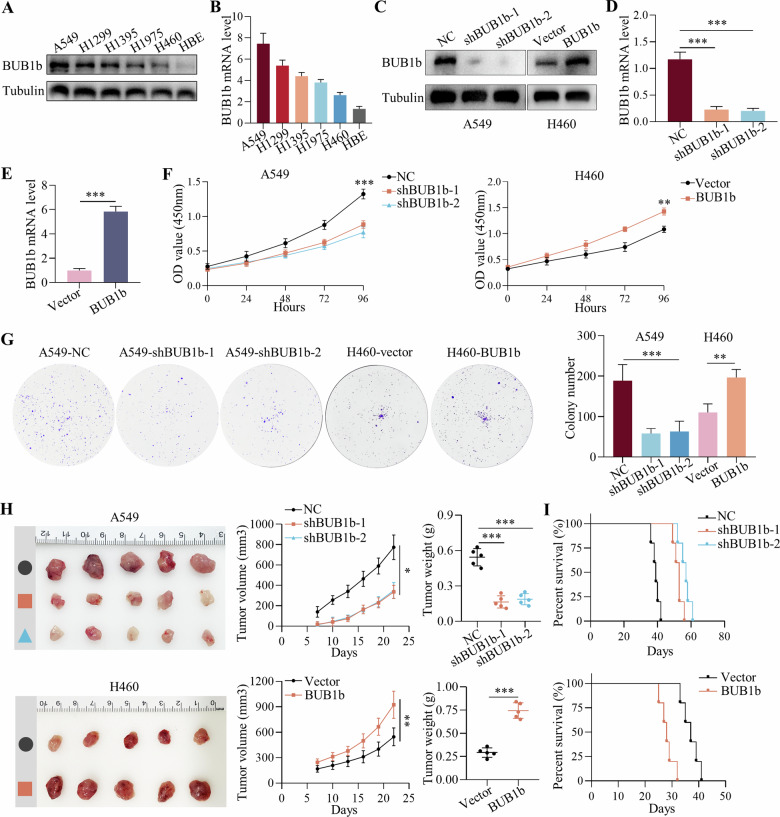
BUB1b regulated the viability of LUAD cells in vitro and in vivo. **A**, **B** The expression of BUB1b in five LUAD cell lines (A549, H1299, H1395, H1975, and H460) and HBE was detected by immunoblotting (**A**); the mRNA level of BUB1b in five LUAD cell lines (A549, H1299, H1395, H1975, and H460) and HBE was detected via qRT-PCR (**B**). **C** The transfection efficiency of lentivirus silencing or overexpressing BUB1b was verified by immunoblotting in A549 and H460 cells. **D** The knockdown of BUB1b in A549 cells with lentivirus was measured by qRT-PCR. **E** The overexpression of BUB1b in H460 cells was confirmed by qRT-PCR. **F**, **G** After the silencing of BUB1b in A549 cells and overexpression of BUB1b in H460 cells, the viability was evaluated by CCK-8 assays (**F**) and colony formation assays (**G**). **H** A549-NC, A549-shBUB1b-1, A549-shBUB1b-2, H460-vector, and H460-BUB1b were subcutaneously implanted into BALB/c nude mice. The tumor size (volume = 1/2 width^2^ × length) was recorded every three days. After the mice were sacrificed, the tumors were resected and weighed. **I** Kaplan‒Meier plots comparing the survival profiles of nude mice bearing tumors derived from A549-NC, A549-shBUB1b-1, A549-shBUB1b-2, H460-vector, and H460-BUB1b cells. Student’s *t*-test and one-way ANOVA were applied to compare the differences. *p < 0.05, **p < 0.01, and ***p < 0.001.

The expression levels of BUB1b were initially evaluated in five LUAD cell lines (A549, H1299, H1395, H1975, and H460) and one bronchial epithelial cell line HBE using immunoblotting and qRT-PCR. Consistently. The results showed that both the protein and mRNA levels of BUB1b were relatively highest in A549 cells and lowest in HBE cells. The expression of BUB1b in HBE was significantly lower than in cancer cell lines (Fig. 2A, B). Thus, BUB1b was silenced in A549 cells and overexpressed in H460 cells transfected with lentiviruses (A549-shBUB1b-1, A549-shBUB1b-2, and A549-BUB1b), and the transfection efficiency was further confirmed (Fig. 2C–E). The CCK-8 assays suggested that viability was impaired by the knockdown of BUB1b, while the overexpression of BUB1b showed the opposite trend (Fig. 2F). Additionally, colony formation assays also demonstrated the oncogenic role of BUB1b (Fig. 2G). To verify the in vitro results, a subcutaneous tumor model in BALB/c nude mice was constructed with the silencing of BUB1b in A549 cells and overexpression in H460 cells. The silencing of BUB1b significantly retarded subcutaneous tumor growth and improved survival, while the overexpression of BUB1b accelerated tumor growth and shortened survival (Fig. 2H–I). Since BUB1b is a mitotic gene, we further verified its function in the cell cycle. Consequently, the overexpression of BUB1b in HBE facilitated the cell cycle progression from G1 to S (Supplementary Fig. 2A, B). Thus, BUB1b could regulate viability and function as an oncogene in LUAD.

### BUB1b conferred ferroptosis resistance to LUAD by targeting NRF2

**Fig. 3:**
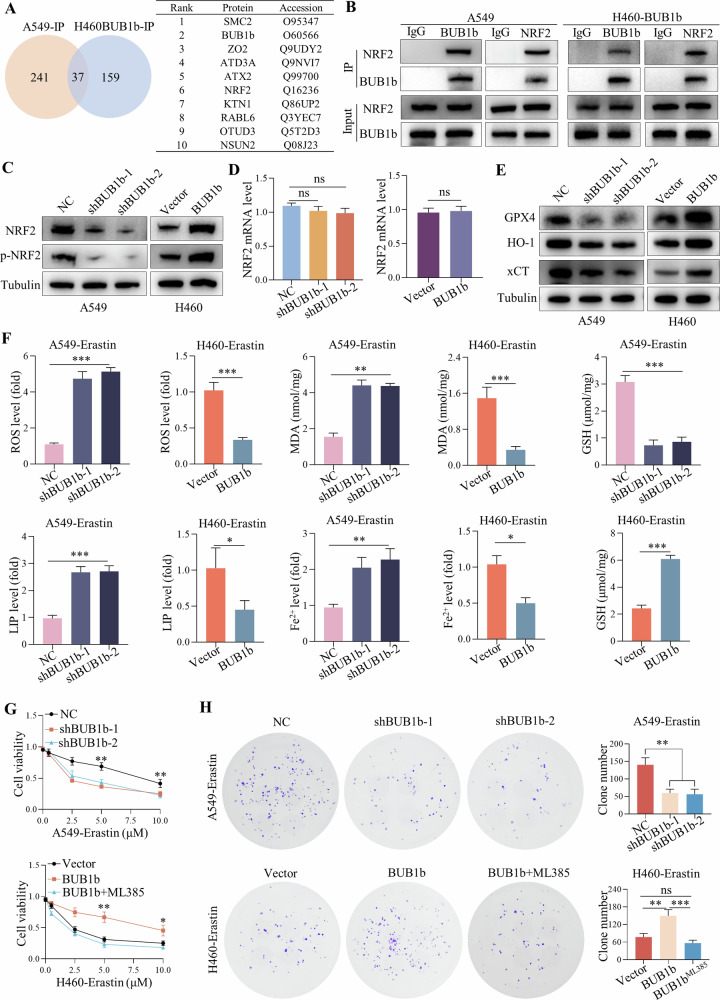
BUB1b conferred ferroptosis resistance to LUAD by targeting NRF2. **A** The immunoprecipitated candidates of A549-IP and H460-BUB1b-IP cells were detected via LC/MS, and the results showing the top ten candidates contained in both A549-IP and H460-BUB1b-IP cells are presented. **B** Co-IP assays were performed in both A549 and H460-BUB1b cells with BUB1b and NRF2 antibodies, respectively. **C**, **D** After knockdown of BUB1b in A549 cells and overexpression of BUB1b in H460 cells using lentivirus, NRF2, and p-NRF2 were detected by immunoblotting (**C**) and qRT-PCR (**D**). **E** After the knockdown of BUB1b in A549 cells and overexpression of BUB1b in H460 cells, NRF2 signaling downstream of SLC7A11, HO-1, and GPX4 was detected by immunoblotting. **F** After the knockdown of BUB1b in A549 cells and overexpression of BUB1b in H460 cells treated with erastin, the levels of ROS, MDA, LIP, Fe^2+^, and GSH were detected via the corresponding detection kits. **G**, **H** The impact of BUB1b knockdown and overexpression in A549 or H460 cells on sensitivity to the ferroptosis inducer erastin was detected via CCK-8 (**G**) and colony formation assays (**H**). The NRF2 inhibitor ML385 was further administered to H460-BUB1b cells. In colony formation assays, the concentration of erastin was 5 μM; Student’s *t*-test and one-way ANOVA were used to compare significant differences. *p < 0.05, **p < 0.01, ***p < 0.001.

To further explore the mechanism by which BUB1b facilitates the progression of LUAD, immunoprecipitation (IP) assays were performed in A549 and BUB1b-overexpressing H460 (H460-BUB1b) cells using a BUB1b antibody. The immunoprecipitated complexes were detected by LC/MS. A total of 278 proteins were detected in A549 IP products, and 196 proteins were found in H460-BUB1b-IP products, of which 37 candidates overlapped in both cell lines (Fig. 3A). Among the top ten candidates in both A549 and H460-BUB1b cells, the transcription factor NRF2, a critical mediator in the ferroptosis pathway, was identified [10]. Thus, it was speculated that BUB1b enhanced the progression of LUAD via interaction with NRF2 and impeded the activation of the ferroptosis pathway. To verify the LC/MS results, immunoprecipitation (Co-IP) assays were performed in both A549 and H460-BUB1b cells with BUB1b and NRF2 antibodies, and BUB1b and NRF2 could interact with each other (Fig. 3B). Moreover, knockdown of BUB1b reduced the protein levels of NRF2 and p-NRF2 in A549 cells, and overexpression of BUB1b in H460 cells elevated the protein expression of NRF2 and p-NRF2, while the transcription level of NRF2 was independent of BUB1b in A549 and H460 cells, which suggested that BUB1b could mediate the stability of NRF2 at the posttranscriptional level (Fig. 3C, D).

The downstream components of NRF2 were also detected, and the immunoblotting results showed that GPX4, xCT, and HO-1 were all downregulated after the silencing of BUB1b or increased in BUB1b-silenced A549 or BUB1b-overexpressing H460 cells (Fig. 3E). In addition, the levels of reactive oxygen species (ROS), malondialdehyde (MDA), labile iron pool (LIP), and Fe^2+^ were increased in A549-shBUB1b cells but decreased in H460-BUB1b cells. Conversely, the antioxidant component glutathione (GSH) was dramatically reduced in A549-shBUB1b cells but increased in H460-BUB1b cells (Supplementary Fig. 3A). Additionally, in the presence of the ferroptosis inducer erastin [11], the changes of ROS, MDA, GSH, LIP, and Fe^2+^ showed the similar trend (Fig. 3F). Knockdown of BUB1b in A549 cells enhanced sensitivity to erastin, while overexpression of BUB1b conferred more resistance to H460 cells, which was further abrogated by administration of the NRF2 inhibitor ML385 (Fig. 3G). In colony formation assays, the knockdown of BUB1b also improved the efficacy of erastin, and ML385 reversed the insensitivity induced by the forced expression of BUB1b (Fig. 3H). We also overexpressed NRF2 in A549-shBUB1b-1 and A549-shBUB1b-2 (Supplementary Fig. 3B). Consequently, NRF2 overexpression enhanced the viability of A549-shBUB1b-1 and A549-shBUB1b-2 (Supplementary Fig. 3C, D). Moreover, enhanced expression of NRF2 could rescue growth inhibition and recover resistance to erastin in A549 with the knockdown of BUB1b (Supplementary Fig. 3E, F). After the treatment of erastin, the level of NRF2 was also increased in H460-BUB1b but downregulated in A549-shBUB1b-1 and A549-shBUB1b-2 (Supplementary Fig. 3G). Thus, BUB1b could confer ferroptosis resistance to LUAD via the stabilization of NRF2 at the posttranscriptional level.

### BUB1b impaired chemotherapy via the inhibition of ferroptosis in LUAD

**Fig. 4:**
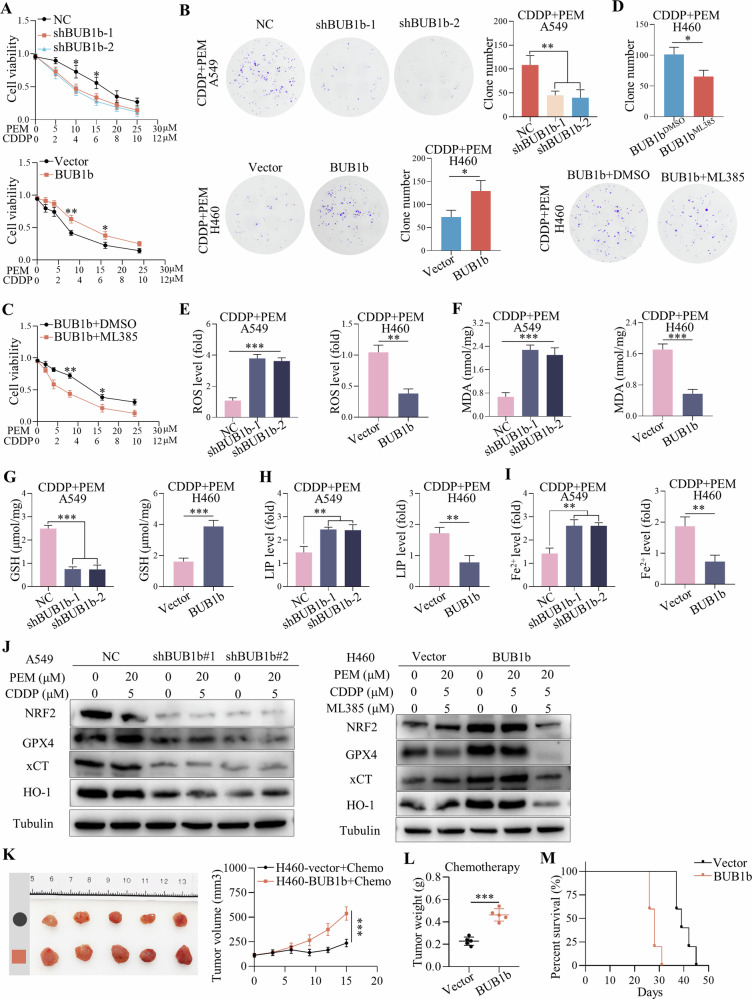
BUB1b enhanced chemoresistance via the inhibition of ferroptosis in LUAD. **A**, **B** The sensitivity to chemotherapy (PEM + CDDP) after the silencing of BUB1b in A549 cells and overexpression of BUB1b in H460 cells was detected via CCK-8 assay (**A**) and colony formation assay (**B**). **C**, **D** The impact of ML385 on the sensitivity to chemotherapy in H460-BUB1b cells was evaluated by CCK-8 assay (**C**) and colony formation assay (**D**). **E–I** The ROS (**E**), MDA (**F**), GSH (**G**), LIP (**H**), and Fe^2+^ (**I**) levels in A549 cells with silenced BUB1b and H460 cells overexpressing BUB1b were measured in a medium containing CDDP (4 μM) and PEM (10 μM). **J** The activation of the NRF2 signaling pathway, including NRF2, GPX4, xCT, and HO-1, was detected by immunoblotting in A549 cells with silenced BUB1b and H460 cells overexpressing BUB1b after treatment with CDDP + PEM. **K** The image of subcutaneous tumors of H460-vector and H460-BUB1b. Chemotherapy was administered when the volume reached 100 mm^3^, and the size was measured every three days for two weeks. **L** The tumor weights of subcutaneous H460-vector and H460-BUB1b tumors treated with chemotherapy were evaluated. **M** Kaplan‒Meier plot presenting the prognosis of nude mice bearing H460-vector and H460-BUB1b tumors treated with chemotherapy. Student’s *t*-test and one-way ANOVA were used to compare the significant differences. *p < 0.05, **p < 0.01, ***p < 0.001.

Previous studies have established a connection between ferroptosis and chemoresistance in various types of cancer [12, 13]. Consequently, we suspected that BUB1b might impair the efficacy of chemotherapy by inhibiting the ferroptosis pathway. In the context of LUAD treatment, cisplatin (CDDP) plus pemetrexed (PEM) is the first-line chemotherapy regimen. The sensitivity to this therapeutic schedule influenced by BUB1b was investigated. Our findings from both the CCK-8 and colony formation assays suggested that knockdown of BUB1b in A549 cells enhanced sensitivity to CDDP/PEM, while overexpression of BUB1b conferred resistance to CDDP/PEM (Fig. 4A, B). This resistance was reversible with the administration of ML385 (Fig. 4C, D). Moreover, overexpression of BUB1b in H460 enhanced the sensitivity to ML385, which explained that it was an NRF2-dependent inhibitor (Supplementary Fig. 4A). In A549 cells, the combination of ML385 and chemotherapy also achieved synergistic effects. Compared with H460 cells which had relatively lower levels of BUB1b, A549 cells were more sensitive to the combination therapy (Supplementary Fig. 4B, C). Thus, BUB1b could confer chemoresistance to LUAD by targeting NRF2. Furthermore, in A549-shBUB1b cells treated with CDDP + PEM, there was a significant increase in ROS, MDA, LIP, and Fe^2+^ levels and a decrease in GSH. In contrast, overexpression of BUB1b in H460 cells showed the opposite trend, with reduced ROS, MDA, LIP, and Fe^2+^ but increased GSH in response to CDDP/PEM (Fig. 4E–I). In H460-BUB1b cells treated with CDDP + PEM and ML385, there was an abrogation of ROS, MDA, LIP, and Fe^2+^ production inhibition but a decrease in the level of GSH (Supplementary Fig. 4D).

Immunoblotting showed the significant inhibition of NRF2 and its downstream targets GPX4, xCT, and HO-1 in A549-shBUB1b cells treated with CDDP/PEM. However, overexpression of BUB1b in H460 cells with CDDP/PEM activated the NRF2 signaling pathway, which was further abolished by ML385 (Fig. 4J). Thus, BUB1b-induced insensitivity to chemotherapy appears to depend on NRF2-mediated ferroptosis resistance. The impact of BUB1b on chemotherapy was further verified in vivo in BALB/c nude mice subcutaneously injected with H460-vector and H460-BUB1b cells. Consistent with the in vitro results, overexpression of BUB1b also impaired the efficacy of chemotherapy on the growth of subcutaneous tumors (Fig. 4K, L) and led to shorter survival (Fig. 4M). We also verified the potential role of BUB1b in chemotherapy via the IHC staining of BUB1b in tumor tissues of LUAD patients accepting neoadjuvant chemotherapy. Consequently, the level of BUB1b was significantly higher in tumor tissues of LUAD patients that had poor response to chemotherapy (Supplementary Fig. 4E).

### Recruitment of OTUD3 by BUB1b stabilized NRF2 in LUAD

**Fig. 5:**
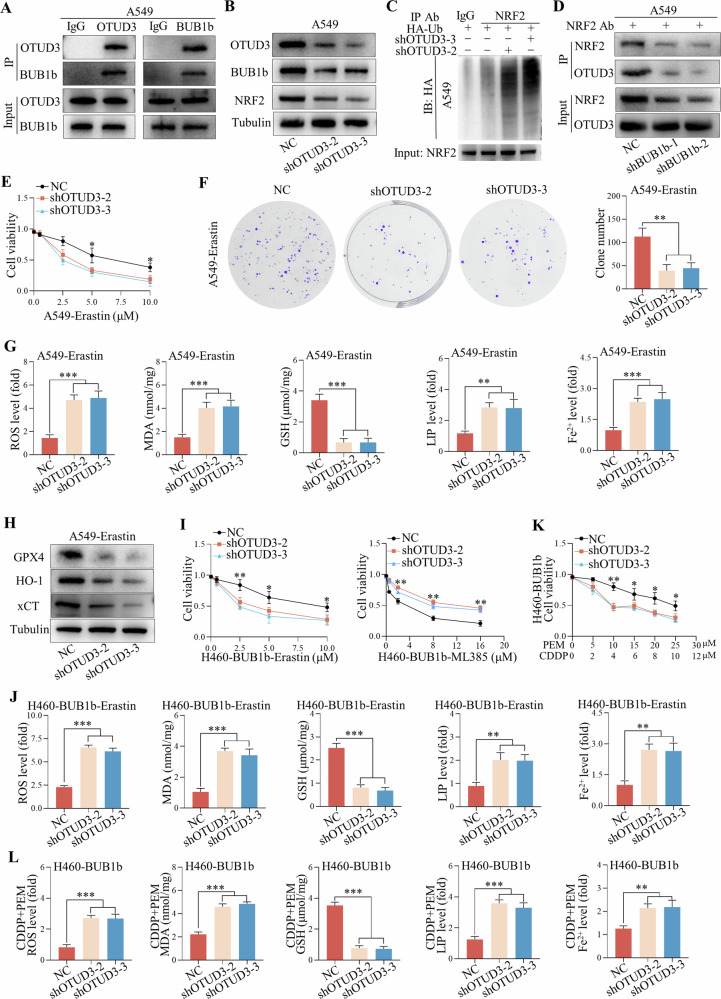
Recruitment of OTUD3 by BUB1b stabilized NRF2 in LUAD. **A** Co-IP assays were performed in A549 using OTUD3 and BUB1b antibodies to verify the interaction of the two proteins. **B** After the knockdown of OTUD3 in A549, BUB1b and NRF2 were detected by immunoblotting. **C** After the knockdown of OTUD3 in A549, ubiquitination level of NRF2 was detected via IP assay. **D** In A549 with the silence of BUB1b, the interaction of NRF2 and OTUD3 was verified via Co-IP assay. **E**, **F** In A549 cells with knockdown of OTUD3, the sensitivity to erastin was verified by CCK-8 assay (**E**) and colony formation assay (**F**). **G** The ROS, MDA, LIP, Fe^2+^, and GSH levels in A549 cells with the silence of OTUD3 were measured after the treatment with erastin. **H** GPX4, HO-1, and xCT were detected in A549 cells by immunoblotting with knockdown of OTUD3 and erastin administration. **I** In H460-BUB1b cells, the sensitivity to erastin and ML385 after the knockdown of OTUD3 cells was evaluated via CCK-8 assay. **J** The ROS, MDA, LIP, Fe^2+^, and GSH levels in H460-BUB1b cells were detected after the silence of OTUD3 and administration of erastin. **K** In H460-BUB1b cells, the impact of OTUD3 knockdown on the sensitivity to PEM + CDDP was evaluated via CCK-8 assay. **L** In H460-BUB1b cells with knockdown of OTUD3, the ROS, MDA, LIP, Fe^2+^, and GSH levels were measured after the treatment with CDDP (4 μM) and PEM (10 μM). One-way ANOVA was performed to compare the differences. *p < 0.05, **p < 0.01, ***p < 0.001.

Since BUB1b mainly functions as a kinase and no evidence of protein stability associated with BUB1b has been reported, we suspected that BUB1b might recruit other proteins to stabilize NRF2, and the immunoprecipitated candidates of both A549-IP and H460BUB1b-IP were reanalyzed. Among the top 10 candidates, OTUD3 was identified as a deubiquitination protein that hindered protein degradation and participated in various signaling pathways [14]. Co-IP assays with OTUD3 and BUB1b antibodies confirmed that OTUD3 and BUB1b could interact with each other (Fig. 5A). Additionally, the knockdown of OTUD3 in A549 cells facilitated the degradation of NRF2 and BUB1b, and the ubiquitination level of NRF2 was obviously enhanced (Fig. 5B, C). The interaction of NRF2 and OTUD3 was further confirmed by Co-IP, and it was shown that NRF2 could form a complex with OTUD3, which was abolished by the knockdown of BUB1b (Fig. 5D). Thus, the interaction of OTUD3 and NRF2 was BUB1b dependent. We also explored the impact of the kinase activity of BUB1b on the recruitment of OTUD3 and NRF2. The domain (766a–1050a) is responsible for the protein kinase in BUB1b and HA-BUB1b^mutant^ (deletion of 766a–1050a) and HA-BUB1b were transfected to H460 cells (Supplementary Fig. 5A). Co-IP results showed that HA-BUB1b^mutant^ could also recruit OTUD3 to stabilize NRF2 (Supplementary Fig. 5B).

In A549 cells, knockdown of OTUD3 sensitized them to the ferroptosis induced by erastin (Fig. 5E). The colony formation results also suggested that viability was significantly impaired in A549 cells with erastin after the silencing of OTUD3 (Fig. 5F). Consistently, the ROS, MDA, LIP, and Fe^2+^ levels were significantly enhanced, while GSH was reduced in A549-shOTUD3-2 and A549-shOTUD3-3 cells treated with erastin (Fig. 5G). Immunoblotting results showed that the knockdown of OTUD3 in A549 cells with erastin decreased the expression of GPX4, HO-1, and xCT (Fig. 5H). To explore whether BUB1b-induced ferroptosis resistance was dependent on OTUD3, OTUD3 was further knocked down in H460-BUB1b cells. Knockdown of OTUD3 rescued the insensitivity to ferroptosis caused by the forced expression of BUB1b but enhanced the resistance to ML385 (Fig. 5I). Consistently, ROS. MDA, LIP, and Fe^2+^ levels were obviously elevated after the silencing of OTUD3, while GSH was reduced after the administration of erastin (Fig. 5J). Moreover, CCK-8 and colony formation assays suggested that OTUD3 knockdown facilitated sensitivity to chemotherapy in H460-BUB1b cells with increased ROS, MDA, LIP, and Fe^2+^ but decreased GSH (Fig. 5K, L and Supplementary Fig. 5C).

### ML385 sensitizes LUAD cells with high BUB1b expression to chemotherapy

**Fig. 6:**
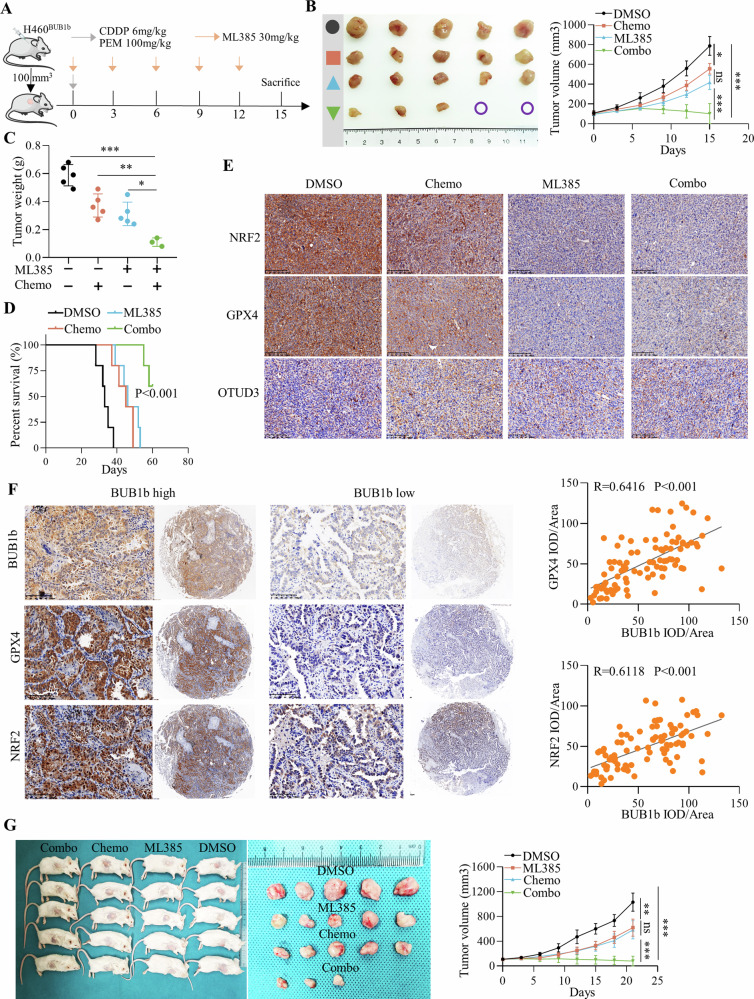
ML385 sensitizes LUAD cells with high BUB1b expression to chemotherapy. **A** Diagram of ML385 and chemotherapy (PEM + CDDP) administration in C57BL/6 mice bearing H460-BUB1b subcutaneous tumors. CDDP (6 mg/kg) and PEM (100 mg/kg) were intraperitoneally injected once and ML385 (30 mg/kg) was intraperitoneally injected at the interval of 3 days when the tumor volume reached 100 mm^3^ and the drug administration lasted for 2 weeks. **B** The image of subcutaneous tumors in DMSO, ML385, chemotherapy, and combo groups and the tumor volume data showing the tumor growth in each group (*n* = 5 for each group). **C** The weight of subcutaneous tumors in DMSO, ML385, chemotherapy, and combo groups. **D** A Kaplan‒Meier plot showed the survival of C57BL/6 mice in DMSO, ML385, chemotherapy, and combo groups, the log-rank test was performed to the survival difference. **E** The represented images of IHC staining of NRF2, GPX4, and OTUD3 in subcutaneous tumors of DMSO, ML385, chemotherapy, and combo groups; one-way ANOVA was performed to compare the significant difference. **F** Representative IHC staining images of BUB1b, NRF2, and GPX4 in the TMA containing 92 tumor tissues and scatter plots showing the correlation of BUB1b with the two proteins. Spearman correlation analysis was performed. **G** The image of PDX subcutaneous tumors in DMSO, ML385, chemotherapy, and combo groups and the tumor volume data showing the tumor growth in each group (*n* = 5 for each group). One-way ANOVA was applied to compare the differences between different groups. *p < 0.05, **p < 0.01, ***p < 0.001.

The above results indicate that BUB1b recruits OTUD3 to stabilize NRF2 and confers ferroptosis resistance, which enhances insensitivity to chemotherapy. Thus, we further explored the synergistic effect of ML385 and chemotherapy in an in vivo setting. The subcutaneous model in BALB/c nude mice was constructed, and CDDP/PEM was administered once when the tumor reached 100 mm^3^, while ML385 was given every three days (Fig. 6A). Compared with either chemotherapy alone or ML385 alone, dual blockade of ML385 and chemotherapy significantly retarded tumor growth, and in two cases, tumor growth was completely diminished after two weeks (Fig. 6B, C). Consistently, dual blockade of ML385 and chemotherapy conferred a greater survival benefit to LUAD with high BUB1b expression in vivo (Fig. 6D). IHC staining showed that the ferroptosis-negative regulators NRF2 and GPX4 were obviously decreased in the chemotherapy + ML385 group (Fig. 6E). Moreover, the relationships among BUB1b, NRF2, and GPX4 were further investigated via IHC staining in a TMA containing 92 LUAD tumor tissues and matched paratumor tissues. The results showed that BUB1b was positively correlated with NRF2 and GPX4 (Fig. 6F). Furthermore, the synergistic effects of ML385 and CDDP/PEM were also investigated in five LUAD patients-derived xenograft models. Similarly, ML385 plus CDDP/PEM significantly impeded tumor growth compared with monotherapy (Fig. 6G).

## Discussion

Various studies have confirmed the aberrant level of BUB1b in different kinds of cancers, including lung cancer. Park et al. identified that a deficiency of acetylation in BUB1b impeded spindle assembly checkpoint signaling and contributed to oncogenesis [15]. The interaction of BUB1b with FOXM1 enhanced radiotherapy resistance in glioblastoma [16]. Additionally, the homozygous mutation in BUB1b resulted in an elevated susceptibility to gastrointestinal oncogenesis [17]. Here, we attempted to uncover the potential mechanism by which BUB1b contributes to the oncogenesis of LUAD. We first confirmed aberrantly highly expressed BUB1b in LUAD tumor tissues compared to normal or para-tumor tissues, which was an independent predictor for a dismal prognosis. Since a previous study identified that BUB1b could bind other proteins to govern oncogenesis, we suspected that this might be an important direction to dissociate the potential mechanism in LUAD. Then, an IP assay was performed, and the immunoprecipitated complexes were detected by LC/MS to identify potential candidates. We found that the recruitment of OTUD3 to NRF2 by BUB1b stabilized the protein level of NRF2 and enhanced resistance to ferroptosis, which conferred chemoresistance to LUAD. Finally, ML385 and chemotherapy achieved synergistic effects in LUAD with high expression of BUB1b.

Ferroptosis is a novel form of cell death first discovered in 2012 that is iron- and ROS-dependent with the characteristics of diminished mitochondrial cristae and ruptured and condensed mitochondrial membranes [18]. Accumulating studies have confirmed the role of ferroptosis in cancer therapy, especially in cancers that are resistant to conventional therapies [19]. SLC7A11, which functions as a cystine/glutamate antiporter, could impede ROS production and abolish p53-mediated tumor suppression [20]. The accumulated lipid peroxides in tumor cells induced by the granules transferred from the neutrophils amplified the necrosis in GBM [21]. Moreover, radiotherapy and IFN-γ achieved synergistic effects via the inhibition of SLC7A11 to enhance lipid oxidation in tumor cells [22]. NRF2 has been confirmed to govern the antioxidant process and maintain redox homeostasis, which can translocate to the nucleus and initiate the transcription of many genes responsible for the prevention of lipid peroxidation [23]. Enzymes related to glutathione synthesis, including SLC7A11 and GSS, are all governed by NRF2 [24]. In addition, NRF2 also regulates iron storage and efflux via the control of FTH1/FTL and SLC40A1 [25]. While BUB1b has been widely studied in various cancers, the potential role of BUB1b in ferroptosis is still elusive. In this study, we found that BUB1b could stabilize NRF2 via the recruitment of OTUD3 and enhance the transcription of downstream GPX4, HO-1, and SLC7A11 to inactivate ferroptosis in LUAD.

PEM plus CDDP is still the first-line chemotherapy for patients with LUAD. However, chemoresistance is still inevitable and limits the therapeutic efficacy of LUAD [26]. Thus, the potential mechanisms governing chemoresistance in LUAD should be further uncovered. To date, DNA damage repair, increased levels of GSH, and mutations associated with the cell cycle have been found to promote chemoresistance. Accumulating studies have reported the association between ferroptosis and chemoresistance. It has been reported that MTTP derived from the exosome of an adipocyte induces chemoresistance in colorectal cancer via the inactivation of ferroptosis [27]. Moreover, cancer-associated fibroblasts confer chemoresistance to gastric cancer via the secretion of miR-522, which suppresses ALOX15 expression and impedes the accumulation of lipid ROS in tumor cells [28]. In this study, elevated BUB1b inactivated ferroptosis via the stabilization of NRF2 and conferred chemotherapy insensitivity to LUAD. In vivo, results suggested the synergistic effects of the NRF2 inhibitor ML385 and chemotherapy in LUAD with high BUB1b expression. Thus, we provided a novel strategy to sensitize LUAD patients to chemotherapy.

In conclusion, this study uncovered the impact of BUB1b on ferroptosis and chemoresistance in LUAD and provided a novel strategy for sensitizing LUAD patients with high BUB1b expression to chemotherapy. The BUB1b-NRF2-OTUD3 complex enhances the NRF2 signaling pathway, which suppresses ferroptosis and induces chemoresistance.

## Materials and methods

### Cell culture and reagents

A549, H1299, H460, H1395, H1975, and HBE cells were purchased from the cell bank of the Chinese Academy of Sciences. A549 and H460 cells were cultured in DMEM (Gibco, USA), and H1299, H1395, H1975, and HBE cells were cultured in RPMI-1640 (Gibco, USA) with 10% fetal bovine serum (Gibco, USA) at 37 °C and 5% CO_2_. In addition, 1% penicillin-streptomycin (Yeasen, Shanghai) was added to prevent bacterial contamination. A ROS detection kit (Beyotime, China, S0053), malondialdehyde (MDA) detection kit (Beyotime, China, S0131S), Fe^2+^ detection kit (Elabscience, E-BC-F101) and glutathione (GSH) detection kit (Beyotime, China, S0053) were used according to the manufacturer’s protocol.

### Real-time qRT-PCR

RNA in LUAD cells and tissue samples was extracted via an RNA extraction kit (Yeasen, Shanghai, 19211ES60). Then, cDNA was obtained via the Hifair III reverse transcriptase cDNA synthesis kit (Yeasen, China). qRT-PCR with Hieff SYBR Green Master Mix (Yeasen, China) in an applied biosystem (Thermo Fisher, USA) was performed to detect the mRNA levels of BUB1b and OTUD3. All parameters were set according to the manufacturer’s instructions, and GAPDH was selected as the internal control. The primers were as follows: BUB1b, F: 5’-CTTAGGGTGCAGCTGGATGT-3’, R: 5’-ACCCATCCCAGAAGACCTGTA-3’; OTUD3, F: 5’-TAAAGCAGCGGGAAGATTTTGA-3’, R: 5’-TGCGATGTGTAACTCCCTCAC-3’; and GAPDH, F: 5’-TCGGAGTCAACGGATTTGGT-3’, R: 5’-TTCCCGTTCTCAGCCTTGAC-3’.

### Lentivirus transfection

Lentiviruses silencing BUB1b and OTUD3 and lentivirus overexpressing BUB1b were designed and constructed by Genomeditech (Shanghai, China). Transfection of the lentivirus in A549 and H460 cells was performed according to the manufacturer’s protocol, and the transfected cells were selected via puromycin (10 μg/mL) for one week. Western blotting and qRT-PCR were both performed to verify the transfection efficiency. The sequences targeting BUB1b and OTUD3 were as follows: shBUB1b#1, 5’-CCTACAAAGGAGACAACTA-3’; shBUB1b#2, 5’-AGGAACAACCTCATTCTAA-3’; shOTUD3#2, 5’-TGGAAATCAGGGCTTAAAT-3’; and shOTUD3#3, 5’-GAGTTACACATCGCATATC-3’.

### TMA

A total of eight LUAD tissues and matched para-tumor tissues were obtained from patients who underwent radical resection of lung lesions in January 2022, and these were used for western blotting and qRT-PCR to detect the expression of BUB1b. A TMA containing 92 LUAD tumor tissues and matched para-tumor tissues was obtained from patients who underwent lung cancer resection between 2008 and 2010 to measure the levels of BUB1b, OTUD3, NRF2, and GPX4. The histopathology of all tissues was confirmed by two experienced pathologists. The consent forms of all patients were obtained, and the Ethics Committee of Fudan University Zhongshan Hospital approved the human and animal ethics.

### Western blotting

The LUAD cells and tumor tissues were digested by lysis buffer for the WB/IP assay (Yeasen, China) with the proteinase inhibitor cocktail (Yeasen, China). After 30 min of incubation on ice, the sample was centrifuged at 12,000×*g* for 15 min, and the protein concentration in the supernatant was detected by a BCA quantification kit (Yeasen, China). The total protein was separated by SDS-PAGE at 120 V and transferred to PVDF membranes (Millipore, USA). Then, the membrane was incubated with primary antibodies overnight at 4 °C and then HRP-conjugated secondary antibodies for 1 h. The target protein on the membrane was finally developed using the ECL reagent in a Tanon imaging system (Shanghai, China). The primary antibodies were as follows: BUB1b (1:1000), Abcam (ab183496); NRF2 (1:1000), Abcam (ab62352); p-NRF2 (1:1000), Abcam (ab76026); OTUD3 (1:1000), Abcam (ab270959); HO-1 (1:1000), Abcam (ab52947); GPX4 (1:2000), Abcam (ab125066); xCT (1:2000), Abcam (ab175186); and Tubulin (1:2000), Abcam (ab7291).

### IHC

The tissues were dewaxed with dimethylbenzene and rehydrated with 95%, 85 and 75% alcohol. Then, 3% H_2_O_2_ was used to block the tissues, which were further incubated at 100 °C for 20 min with 5% citric acid to accomplish antigen retrieval. Next, bovine serum albumin was added to abrogate nonspecific binding. Subsequently, the tissues were incubated with primary antibody at 4 °C for at least 8 h. Finally, the Streptavidin Peroxidase IHC assay kit (ZSGB-Bio, China) was used to measure the level of the target protein. The expression level was evaluated via Image-Pro Plus 6.0 software and calculated as the average gray value. The evaluation was independently performed by two pathologists. The primary antibodies used in this study were as follows: BUB1b (1:200), Abcam (ab183496); NRF2 (1:100), Abcam (ab62352); OTUD3 (1:200), Abcam (ab270959); and OTUD3 (1:300), Abcam (ab270959).

### Colony formation assay

A549 and H460 cells were digested and suspended in DMEM. After cell counting, approximately 1000 cells per well were added to the 6-well plate. Then, these cells were suspended in DMEM with 10% FBS and different concentrations of erastin or ML385 for 2 weeks. When each colony contained at least 50 cells, the plates were fixed with 4% paraformaldehyde for 10 min and stained with 0.1% crystal violet for 20 min.

### Iron assay

The intracellular level of the LIP was measured via calceinacetoxymethyl ester and DFO. Cells were firstly incubated with calcein (2 μmol/L) for 0.5 h and iron was retracted from calcein using DFO (100 μmol/L). The fluorescence was detected at 485 nm/535 nm and the gap between the fluorescence intensity with or without DFO was quantified as the level of LIP.

### CCK-8 assay

To assess viability, approximately 1000 cells were seeded in each well of a 96-well plate. After 12 h, the supernatant was removed, and the cells were cultured with DMEM containing 10% FBS and 10% CCK8 at a volume of 100 μL/well and incubated for 2 h. The optical density (OD) value was measured every 24 h for 4 days, with a final measurement at 450 nm. To evaluate sensitivity to erastin and ML385, approximately 1500 cells were added to each well of a 96-well plate and cultured in DMEM with 10% FBS (100 μL/well). After a 24-h incubation, the supernatant was replaced with DMEM containing 10% FBS and varying concentrations of erastin and ML385 for three days. Finally, the OD value at 450 nm was detected.

### Co-IP and ubiquitination assay

A549 and H460-BUB1b cells (2 × 10^7^) were lysed with IP lysis buffer (Beyotime, Shanghai) on ice for 30 min with protease inhibitor (Yeasen, China) and then centrifuged at 12,000×*g* for 15 min. Subsequently, the supernatants were incubated with 2.5 μg of either anti-IgG or anti-BUB1b overnight at 4 °C. This was followed by further incubation with 20 μL of protein A/G beads (MedChemExpress, USA) for 6 h at 4 °C. The beads were separated from the supernatant using a magnetic separator and washed three times with PBS. Finally, the beads were resuspended in 60 μL of lysis buffer and boiled at 100 °C for 10 min, and the resultant supernatant containing immunoprecipitated complex was used for mass spectrometry and western blotting. For the NRF2 ubiquitination assay, A549-NC, A549-shOTUD3-2, and A549-shOTUD3-3 cells were transfected with HA-ubiquitin using a calcium phosphate transfection kit (Yeasen, Shanghai) for 24 h. These cells were then incubated with the proteasome inhibitor MG132 (20 μM) for 8 h. The pretreated cells were lysed with IP buffer, incubated with NRF2 antibody overnight, and then with A/G beads for 6 h at 4 °C. Finally, the beads were washed three times with PBS, and the immunoprecipitated proteins were separated from the beads by boiling. The ubiquitination level of NRF2 was detected via western blotting using an HA antibody.

### Xenograft mouse model and drug administration

BALB/c nude and NSG mice aged 4–6 weeks were purchased from Charles River (Beijing, China) and raised in a pathogen-free environment to establish the xenograft model. Specifically, A549-NC/shBUB1b-1/shBUB1b/2 and H460-vector/BUB1b (5 × 10^6^ cells) were injected into the right flank of nude mice in a volume of 200 μL (*n* = 5 for each group). The length and width of the subcutaneous tumors were measured daily, and the volume was calculated as follows: volume = ½ length × width^2^. After 28 days, tumors were excised from the anesthetized mice, weighed, and used for IHC staining. To verify the synergistic effects of chemotherapy and ML385 in vivo, H460-BUB1b cells were injected into the right flank of nude mice. When the tumor volume reached approximately 100 mm^3^, CDDP (6 mg/kg) and PEM (100 mg/kg) were administered intraperitoneally once, and ML385 (30 mg/kg) was administered every three days for 2 weeks. Mice that failed to construct the subcutaneous tumor were excluded. All mice were randomly allocated for each group. No blinding of the animal experiment was done. Further verification in the patient-derived xenograft model (PDX) was described as follows: The tumor tissues collected from 5 LUAD patients were cut into small pieces and implanted into the right flank of NSG mice. When the tumor volume reached approximately 100 mm^3^, CDDP (6 mg/kg) and PEM (100 mg/kg) were administered intraperitoneally once, and ML385 (30 mg/kg) was administered every three days for 3 weeks. The Animal Committee of Zhongshan Hospital approved the protocol of this study.

### Statistical analysis

SPSS 23.0 (IBM, USA) and GraphPad Prism 8 (GraphPad Software, USA) were used to perform statistical analysis. All data are presented as the mean ± standard deviation (SD) based on at least three independent replicates. Student’s *t*-test and chi-square test were used for comparisons between the two groups. Kruskal–Wallis one-way ANOVA followed by Dunn’s multiple comparison tests was applied for the comparison of at least three groups. Multistep Cox regression analysis was performed to identify predictors of a dismal prognosis in LUAD patients. Kaplan–Meier and log-rank tests were used for the comparison of survival differences. Spearman’s correlation analysis was performed to identify the correlation coefficients of BUB1b, NRF2, and OTUD3. All results were two-sided, and *p* < 0.05 was regarded as statistically significant.

### Supplementary information

Supplementary figure 1

Supplementary figure 2

Supplementary figure 3

Supplementary figure 4

Supplementary figure 5

Supplementary legends

The clinical information of 92 patients with LUAD

Original full-length western blots

## Data Availability

No primary data sets have been generated and deposited. An expanded view of this article is available online.
