# Supplementary figures and images for: BUB1b impairs chemotherapy sensitivity via resistance to ferroptosis in lung adenocarcinoma
Source: Cell Death Dis. 2024 Jul 23;15(7):525. doi: 10.1038/s41419-024-06914-0 (PMC11266579; doi:10.1038/s41419-024-06914-0)

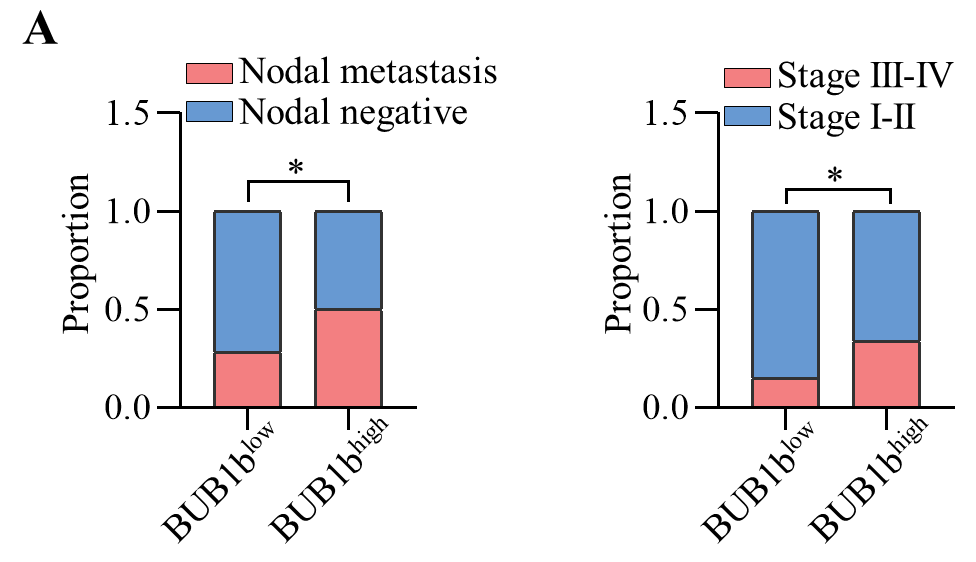

Supplement: Supplementary file 1 — Supplementary figure 1 [file 41419_2024_6914_MOESM1_ESM.tif]

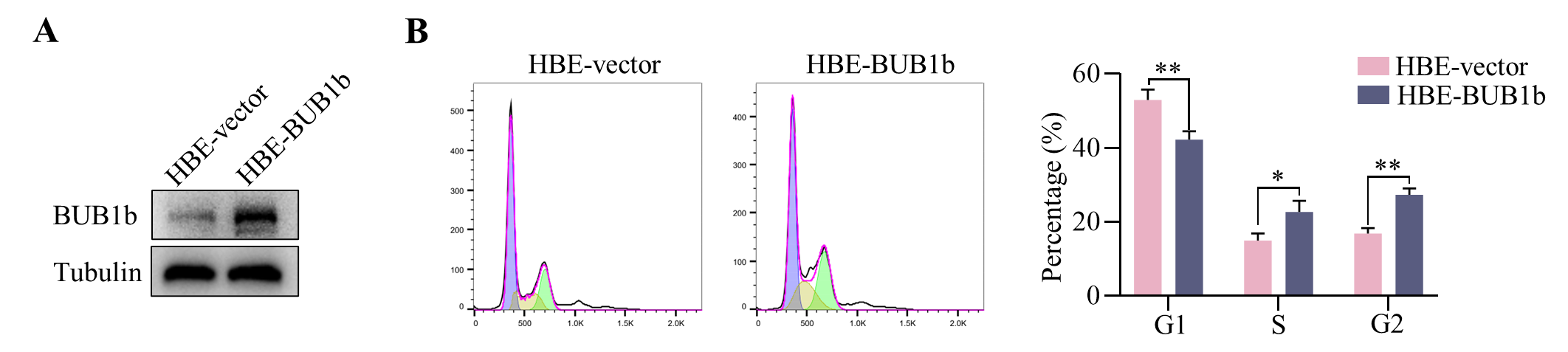

Supplement: Supplementary file 2 — Supplementary figure 2 [file 41419_2024_6914_MOESM2_ESM.tif]

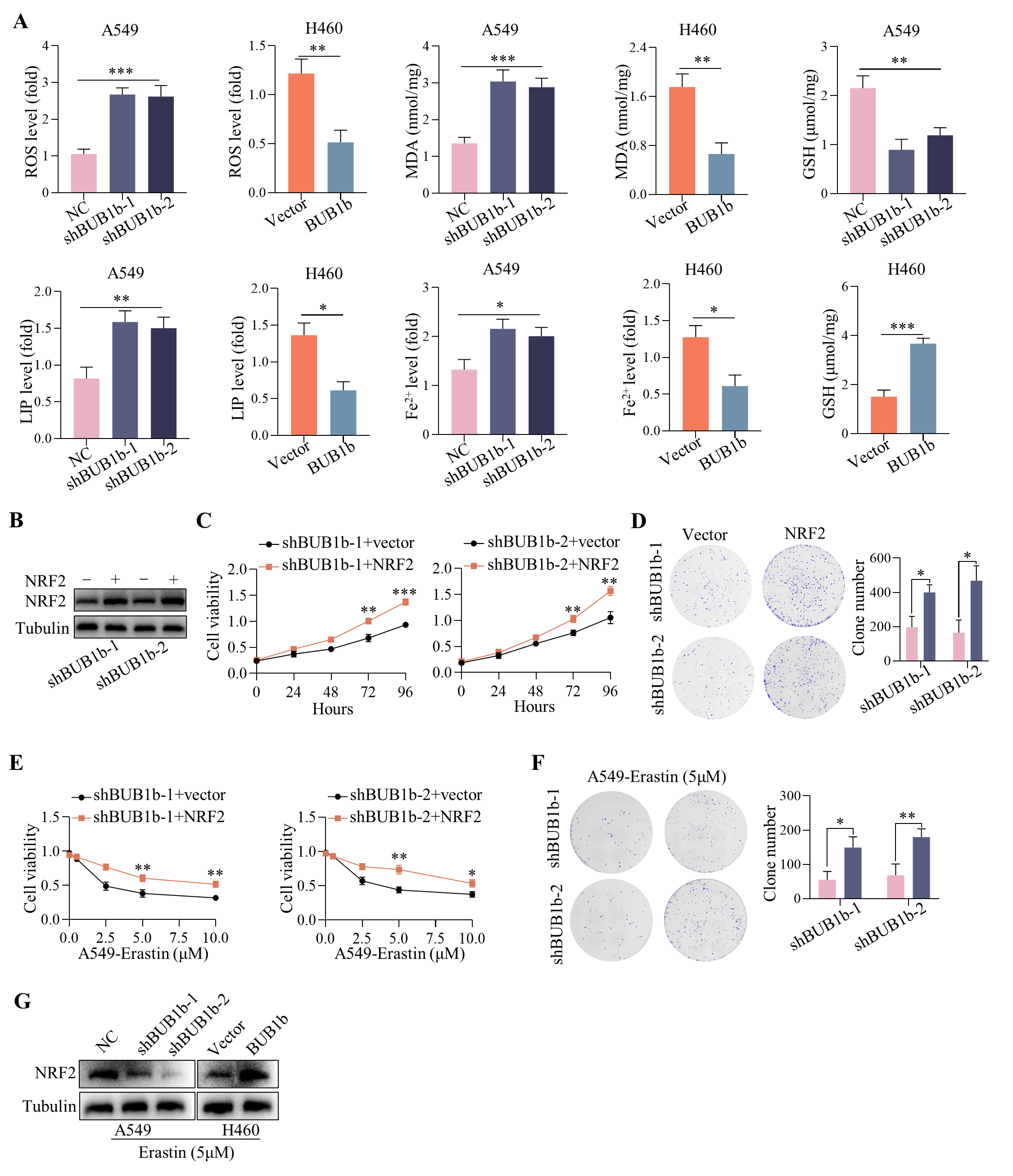

Supplement: Supplementary file 3 — Supplementary figure 3 [file 41419_2024_6914_MOESM3_ESM.tif]

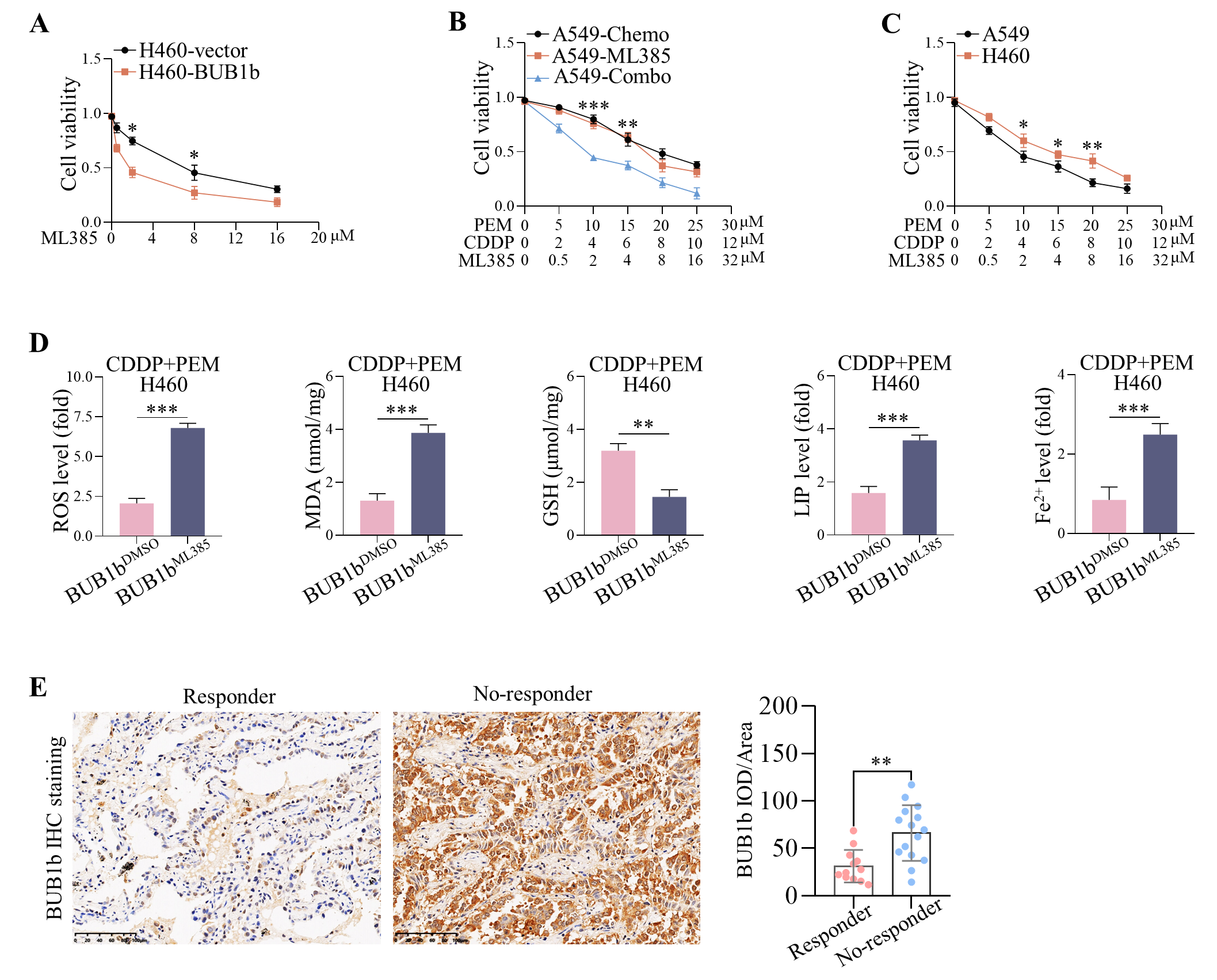

Supplement: Supplementary file 4 — Supplementary figure 4 [file 41419_2024_6914_MOESM4_ESM.tif]

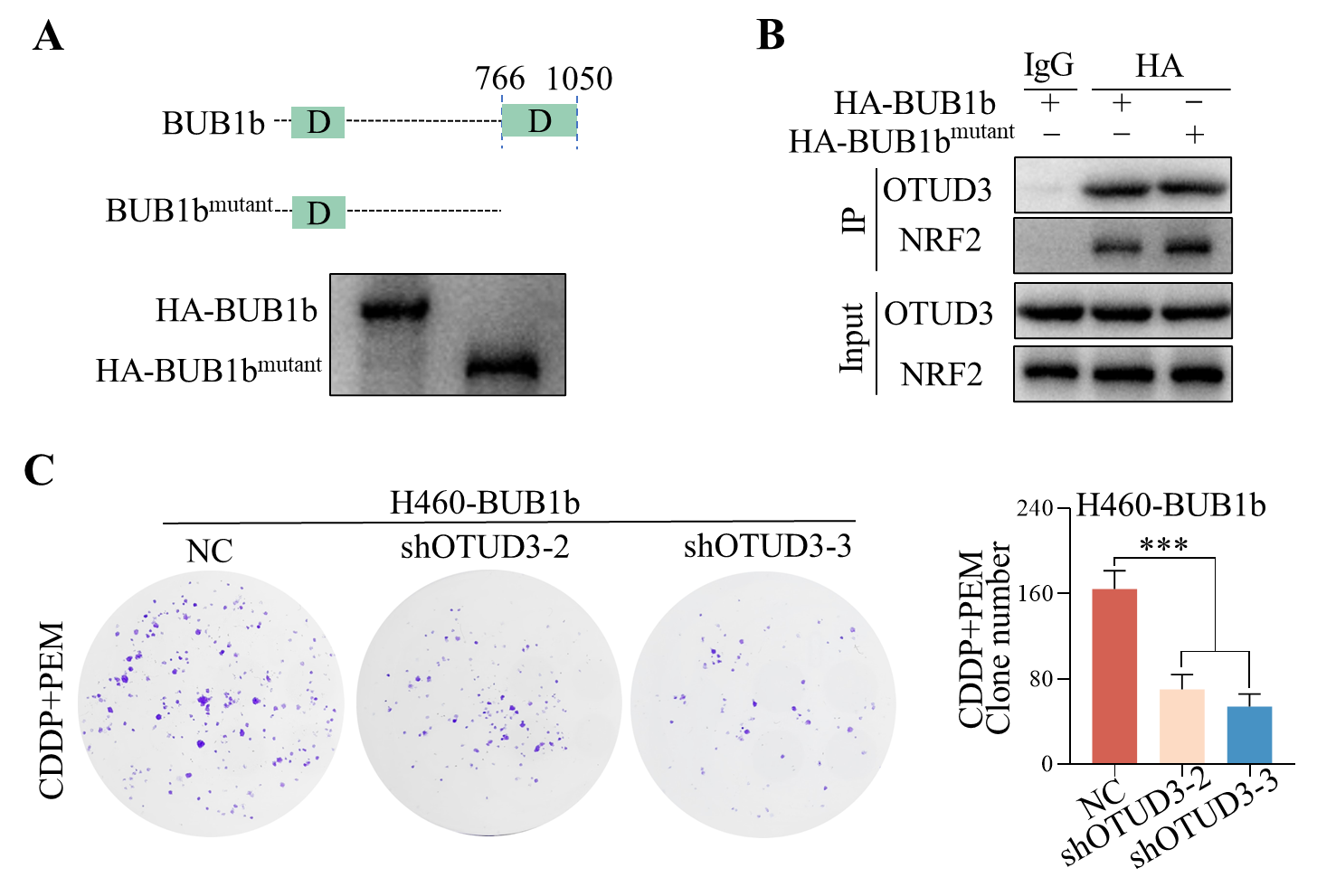

Supplement: Supplementary file 5 — Supplementary figure 5 [file 41419_2024_6914_MOESM5_ESM.tif]
