# Supplementary material for: BUB1b impairs chemotherapy sensitivity via resistance to ferroptosis in lung adenocarcinoma
Source: Cell Death Dis. 2024 Jul 23;15(7):525. doi: 10.1038/s41419-024-06914-0 (PMC11266579; doi:10.1038/s41419-024-06914-0)
Supplement: Supplementary file 6 — Supplementary legends [file 41419_2024_6914_MOESM6_ESM.docx]

**Supplementary Fig. 1**

**A**. The proportion of nodal metastasis, nodal negative, TNM stage I-II and III-IV in BUB1b^low^ and BUB1b^high^. Chi-square test was applied. * P < 0.05.

**Supplementary Fig. 2**

**A**. The overexpression of BUB1b in HBE was measured via immunoblotting; **B**. HBE-vector and HBE-BUB1b were stained with PI and the cell cycle was detected via flow cytometry. Statistical difference was calculated using Student’s t test. * P < 0.05, ** P < 0.01.

**Supplementary Fig. 3**

**A**. After knockdown of BUB1b in A549 cells and overexpression of BUB1b in H460 cells treated with erastin, the levels of ROS, MDA, LIP, Fe^2+^ and GSH were detected; **B**. The overexpression of NRF2 in A549-shBUB1b-1 and A549-shBUB1b-2 were detected via immunoblotting; **C**, **D**. The impact of NRF2 overexpression on the viability of A549-shBUB1b-1 and A549-shBUB1b-2 was measured by CCK-8 (**C**) and colony formation assay (**D**); **E**, **F**. The impact of NRF2 overexpression on the sensitivity to ferroptosis induced by Erastin was evaluated by CCK-8 (**E**) and colony formation assay (**F**). **G**. After Erastin treatment, the level of NRF2 in H460 overexpressing BUB1b and A549 with BUB1b knockdown was investigated via immunoblotting. Statistical difference was evaluated using Student’s t test. * P < 0.05, ** P < 0.01.

**Supplementary Fig. 4**

**A**. The impact of BUB1b overexpression on the sensitivity to ML385 in H460 cells were evaluated using CCK-8 assay; **B**. The synergistic effects of ML385 and chemotherapy in A549 cells were measured via CCK-8 assay; **C**. The sensitivity of A549 and H460 cells to the combination of ML385 and chemotherapy was measured via CCK-8 assay; **D**. After administration of ML385, the ROS, MDA, LIP, Fe^2+^ and GSH in H460-BUB1b treated with chemotherapy were measured; **E**. IHC staining of BUB1b in the tumor tissues of LUAD patients accepting neoadjuvant chemotherapy. Statistical difference was evaluated using Student’s t test. * P < 0.05, ** P < 0.01, *** P < 0.001.

**Supplementary Fig. 5**

**A**. A sketch map of BUB1b mutation with the deletion of a domain (766a-1050a) responsible for protein kinase; **B**. Co-IP assay with the HA antibody was performed in H460 cells transfected with HA-BUB1b and HA-BUB1b^mutant^; **C**. In H460-BUB1b cells, the impact of OTUD3 knockdown on the sensitivity to PEM+CDDP was evaluated via colony formation assay. Statistical difference was evaluated using one-way ANOVA test. *** P < 0.001
