# Supplementary material for: BUB1b impairs chemotherapy sensitivity via resistance to ferroptosis in lung adenocarcinoma
Source: Cell Death Dis. 2024 Jul 23;15(7):525. doi: 10.1038/s41419-024-06914-0 (PMC11266579; doi:10.1038/s41419-024-06914-0)
Supplement: Supplementary file 7 — The clinical information of 92 patients with LUAD [file 41419_2024_6914_MOESM7_ESM.docx]

Supplementary table 1:

| Variables |  | BUB1b expression | |
| --- | --- | --- | --- |
|  |  | low | high |
| Sex |  |  |  |
|  | Male | 21 | 17 |
|  | Female | 25 | 29 |
| Age (years) |  |  |  |
|  | ≤ 60 | 32 | 29 |
|  | > 60 | 14 | 17 |
| Smoking |  |  |  |
|  | Yes | 16 | 23 |
|  | No | 30 | 23 |
| Tumor size (cm) |  |  |  |
|  | <3 | 32 | 23 |
|  | >3 | 14 | 23 |
| Differentiation |  |  |  |
|  | II | 17 | 14 |
|  | II-III | 29 | 32 |
| Lymph metastasis |  |  |  |
|  | No | 34 | 20 |
|  | Yes | 14 | 24 |
| TNM |  |  |  |
|  | I-II | 41 | 29 |
|  | III-IV | 7 | 15 |

The clinical information of 92 patients with LUAD
