# Supplementary material for: BUB1b impairs chemotherapy sensitivity via resistance to ferroptosis in lung adenocarcinoma
Source: Cell Death Dis. 2024 Jul 23;15(7):525. doi: 10.1038/s41419-024-06914-0 (PMC11266579; doi:10.1038/s41419-024-06914-0)
Supplement: Supplementary file 8 — Original full-length western blots [file 41419_2024_6914_MOESM8_ESM.pptx]

## Slide 1
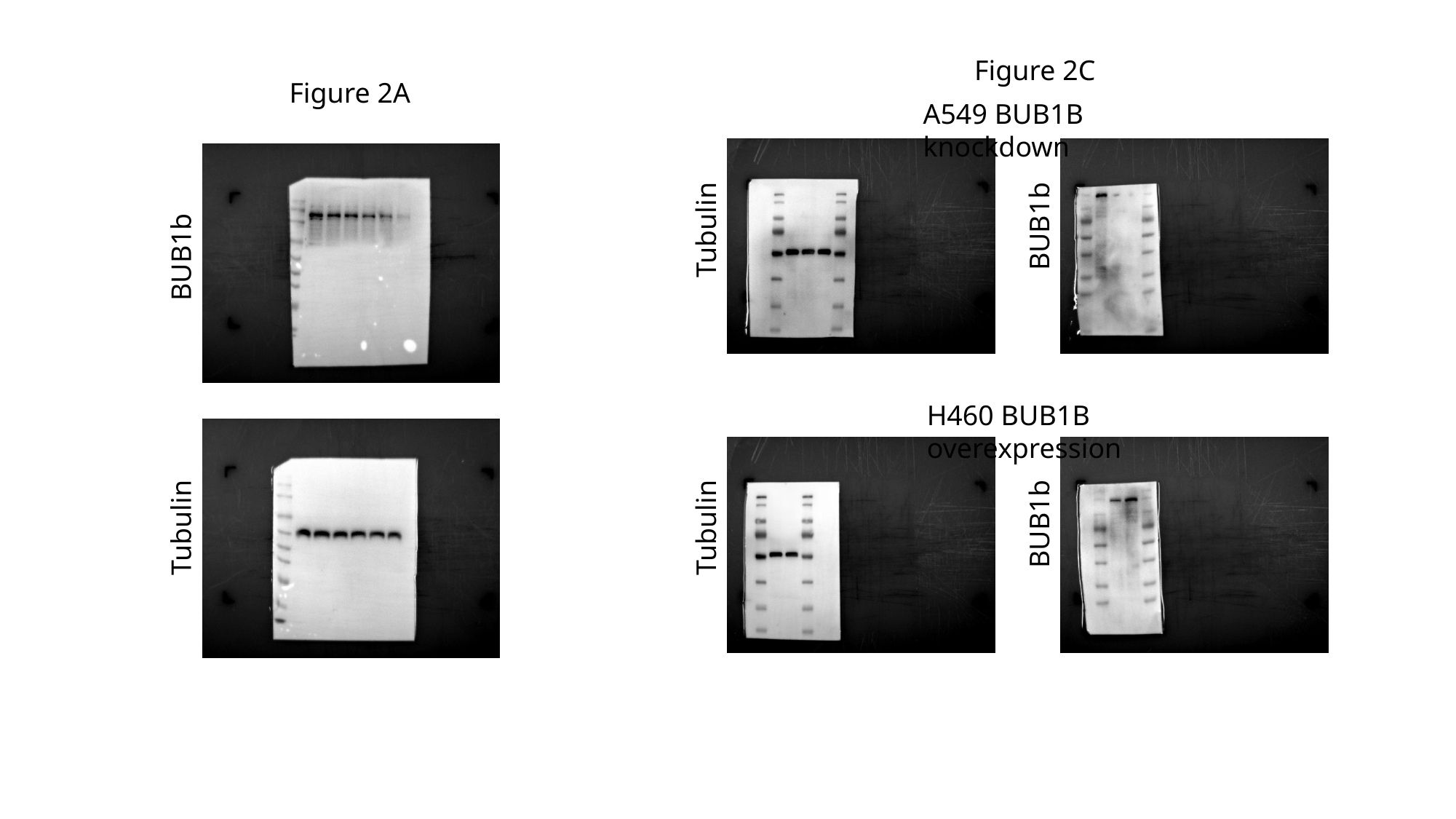

Figure 2C
Figure 2A
A549 BUB1B knockdown
BUB1b
Tubulin
BUB1b
H460 BUB1B overexpression
BUB1b
Tubulin
Tubulin

## Slide 2
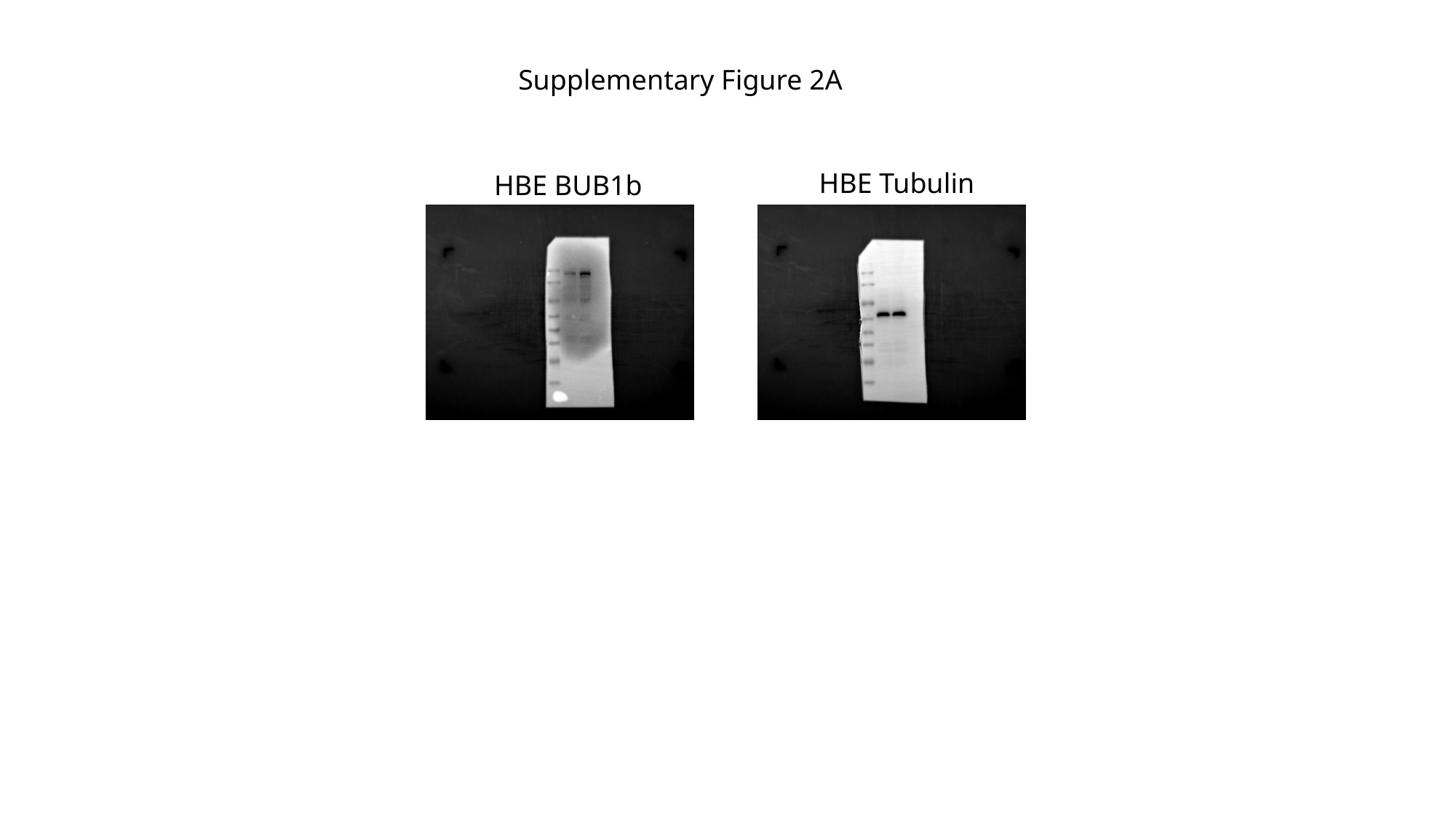

Supplementary Figure 2A
HBE Tubulin
HBE BUB1b

## Slide 3
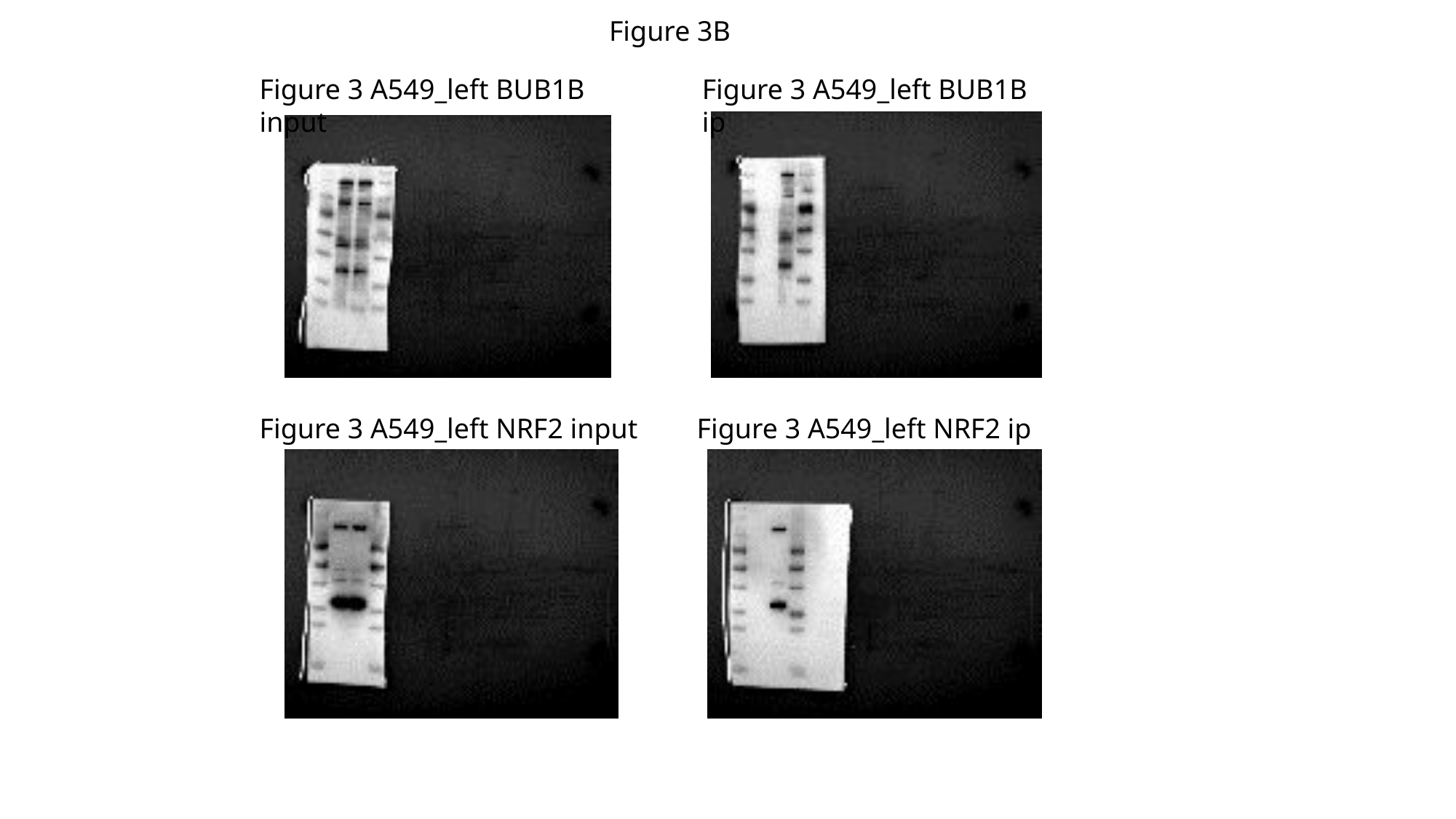

Figure 3B
Figure 3 A549_left BUB1B input
Figure 3 A549_left BUB1B ip
Figure 3 A549_left NRF2 input
Figure 3 A549_left NRF2 ip

## Slide 4
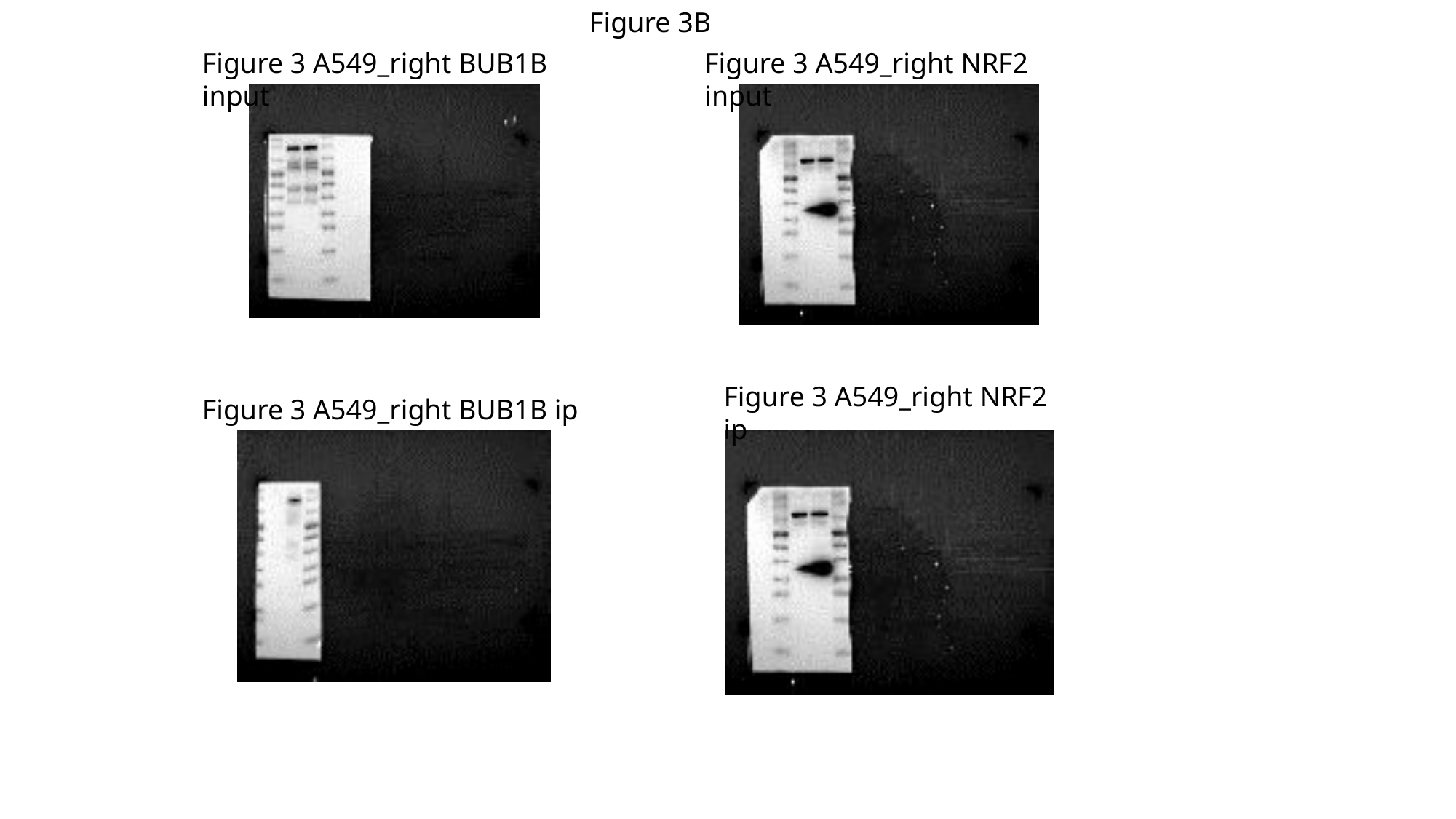

Figure 3B
Figure 3 A549_right BUB1B input
Figure 3 A549_right NRF2 input
Figure 3 A549_right NRF2 ip
Figure 3 A549_right BUB1B ip

## Slide 5
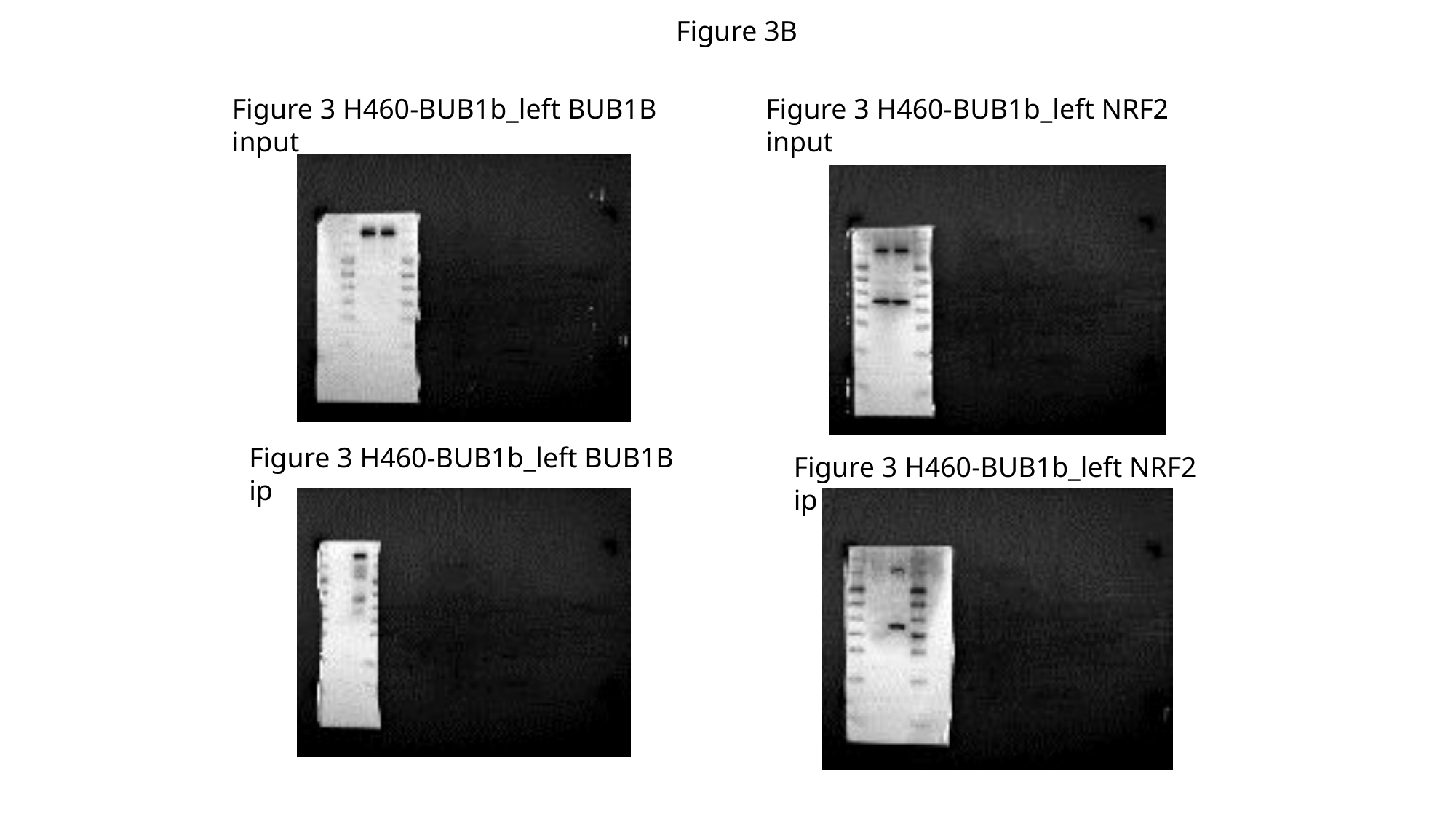

Figure 3B
Figure 3 H460-BUB1b_left BUB1B input
Figure 3 H460-BUB1b_left NRF2 input
Figure 3 H460-BUB1b_left BUB1B ip
Figure 3 H460-BUB1b_left NRF2 ip

## Slide 6
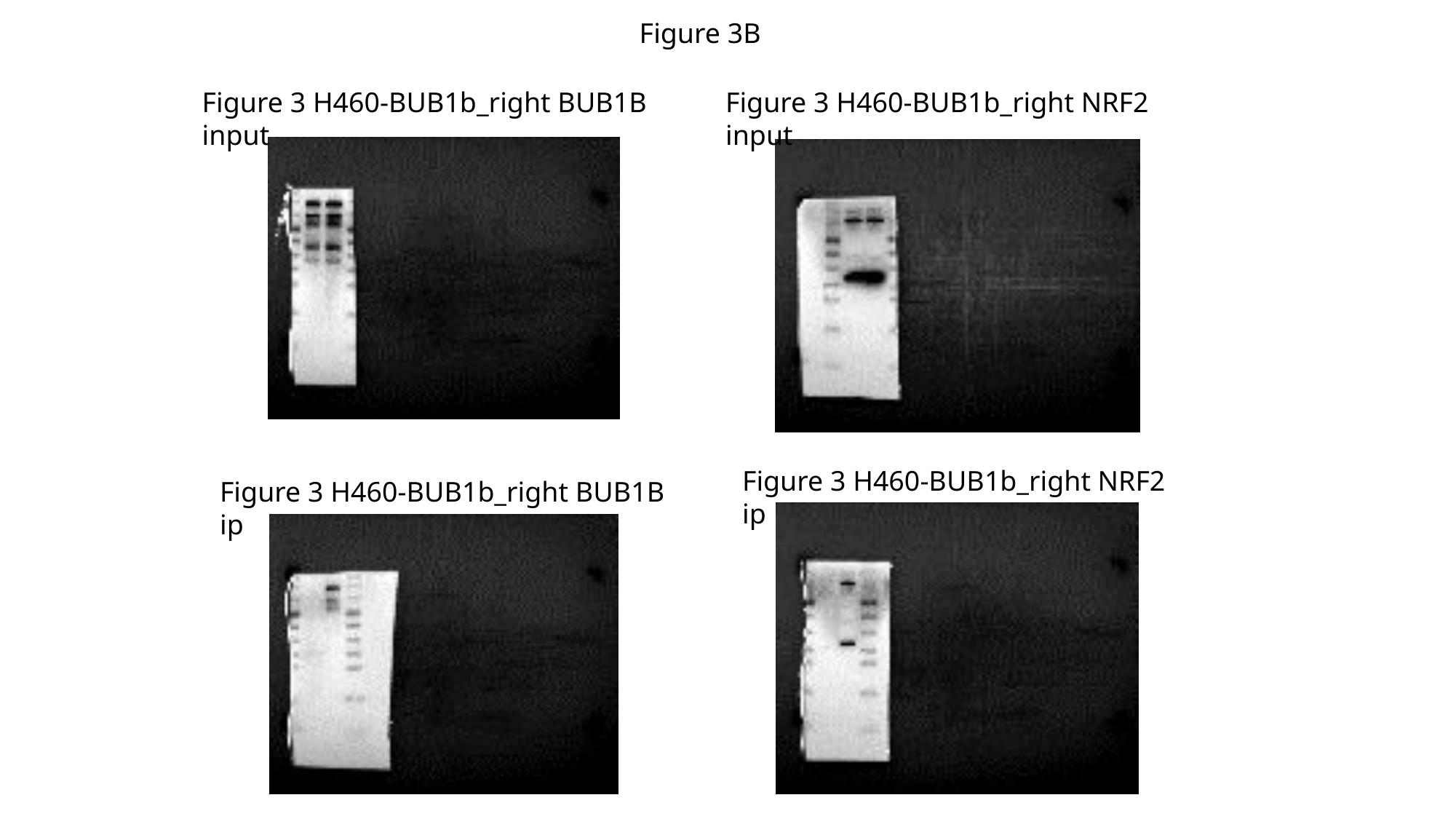

Figure 3B
Figure 3 H460-BUB1b_right BUB1B input
Figure 3 H460-BUB1b_right NRF2 input
Figure 3 H460-BUB1b_right NRF2 ip
Figure 3 H460-BUB1b_right BUB1B ip

## Slide 7
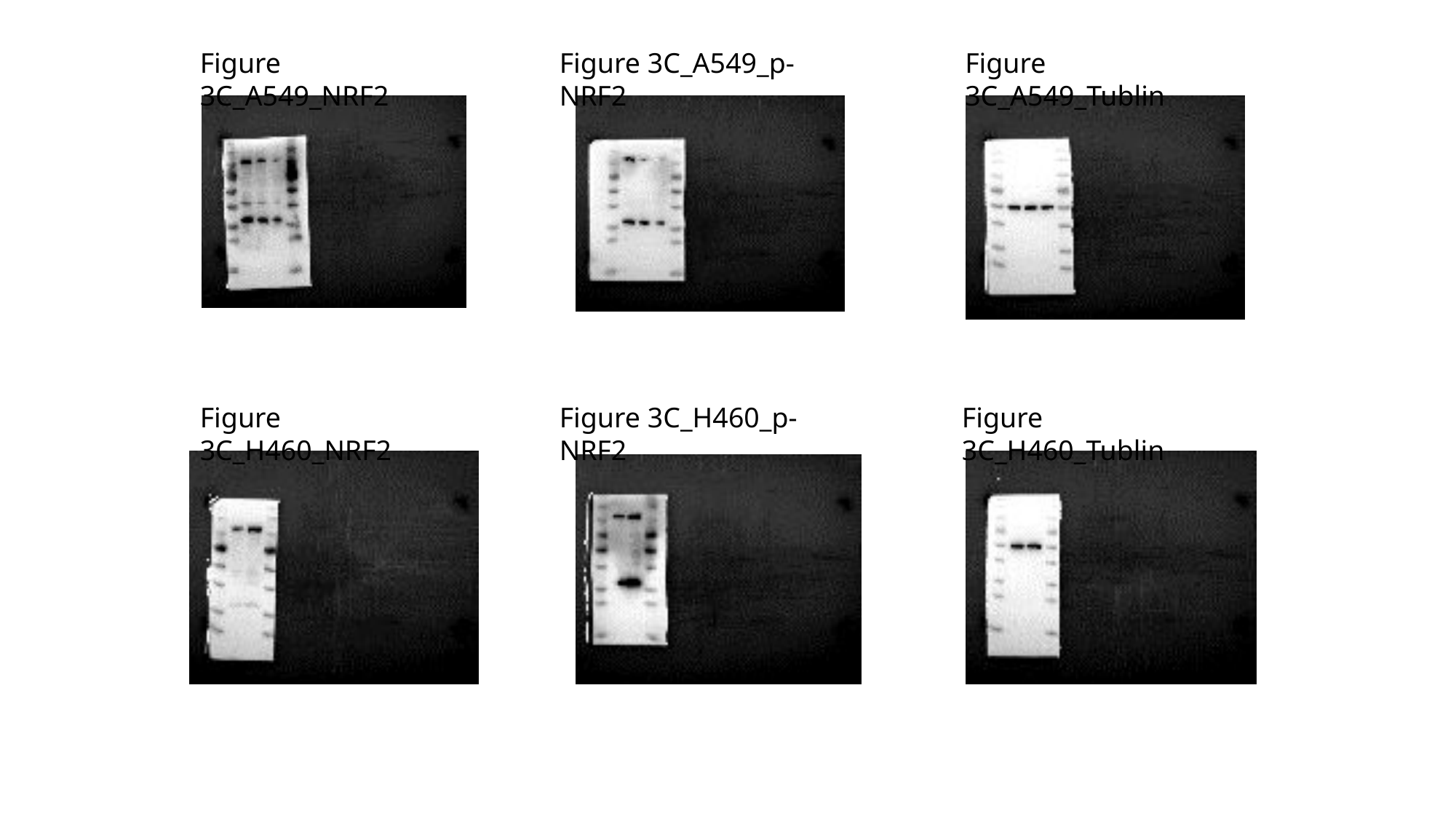

Figure 3C_A549_NRF2
Figure 3C_A549_p-NRF2
Figure 3C_A549_Tublin
Figure 3C_H460_NRF2
Figure 3C_H460_p-NRF2
Figure 3C_H460_Tublin

## Slide 8
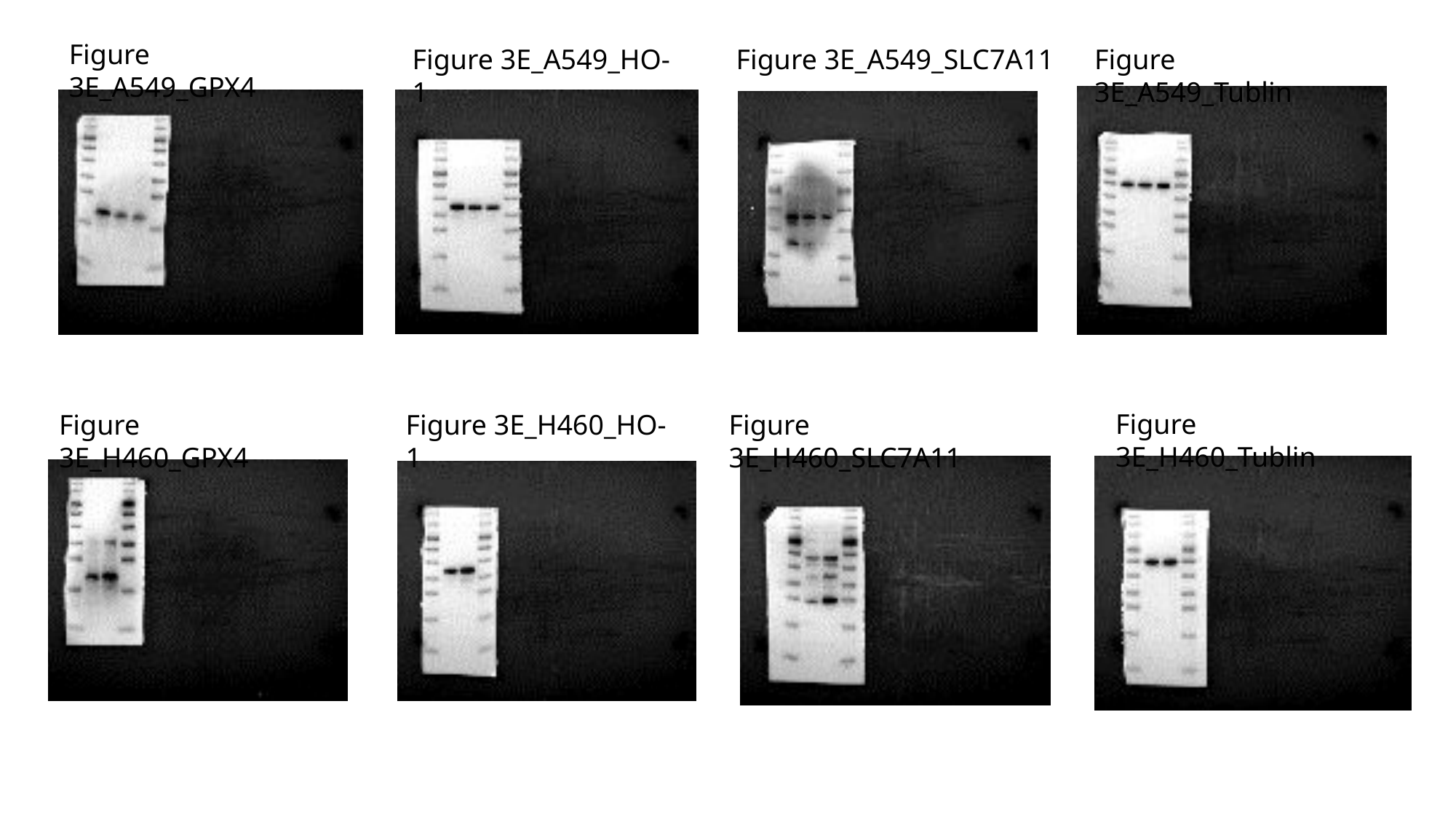

Figure 3E_A549_GPX4
Figure 3E_A549_HO-1
Figure 3E_A549_SLC7A11
Figure 3E_A549_Tublin
Figure 3E_H460_Tublin
Figure 3E_H460_GPX4
Figure 3E_H460_HO-1
Figure 3E_H460_SLC7A11

## Slide 9
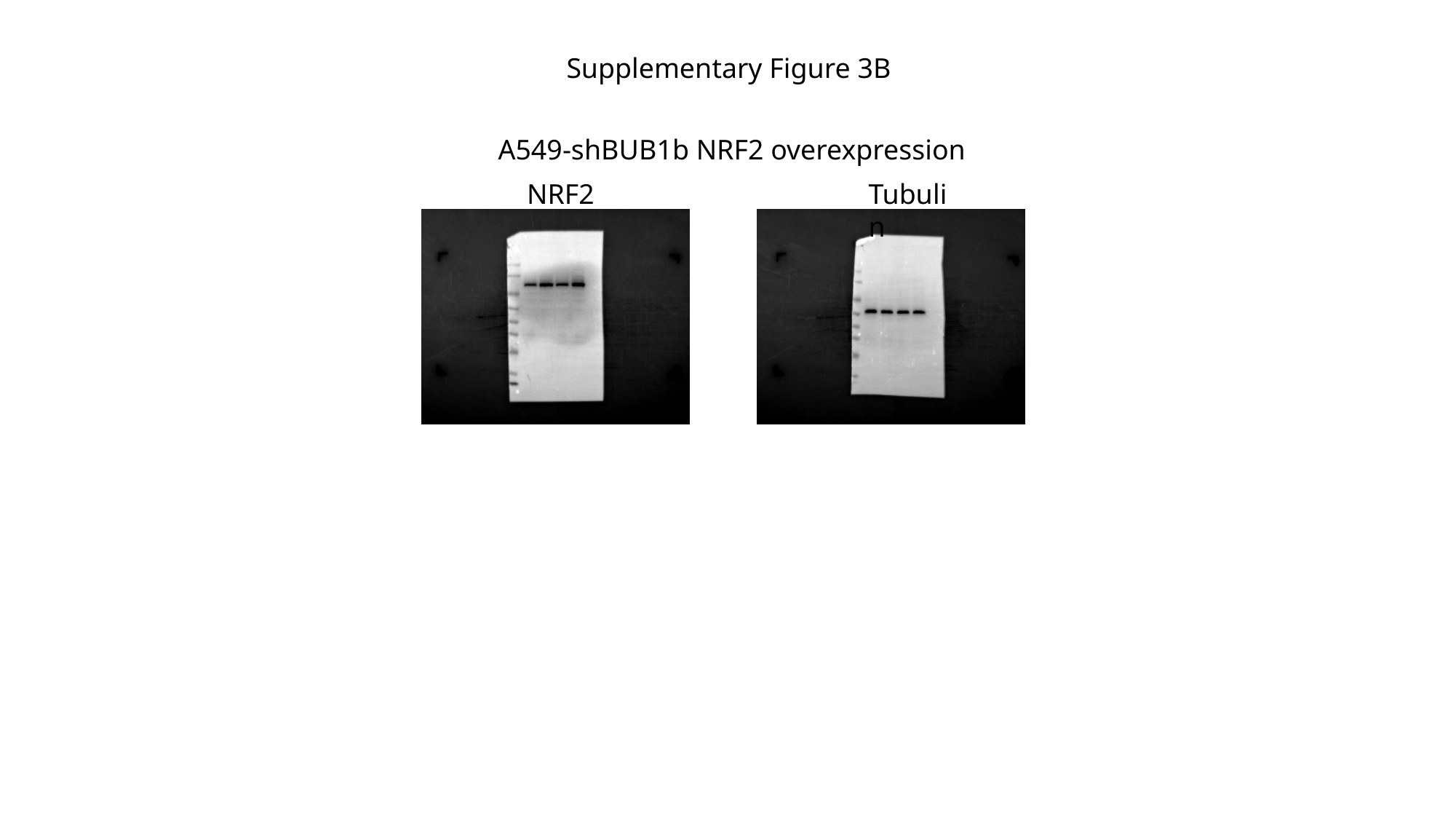

Supplementary Figure 3B
A549-shBUB1b NRF2 overexpression
NRF2
Tubulin

## Slide 10
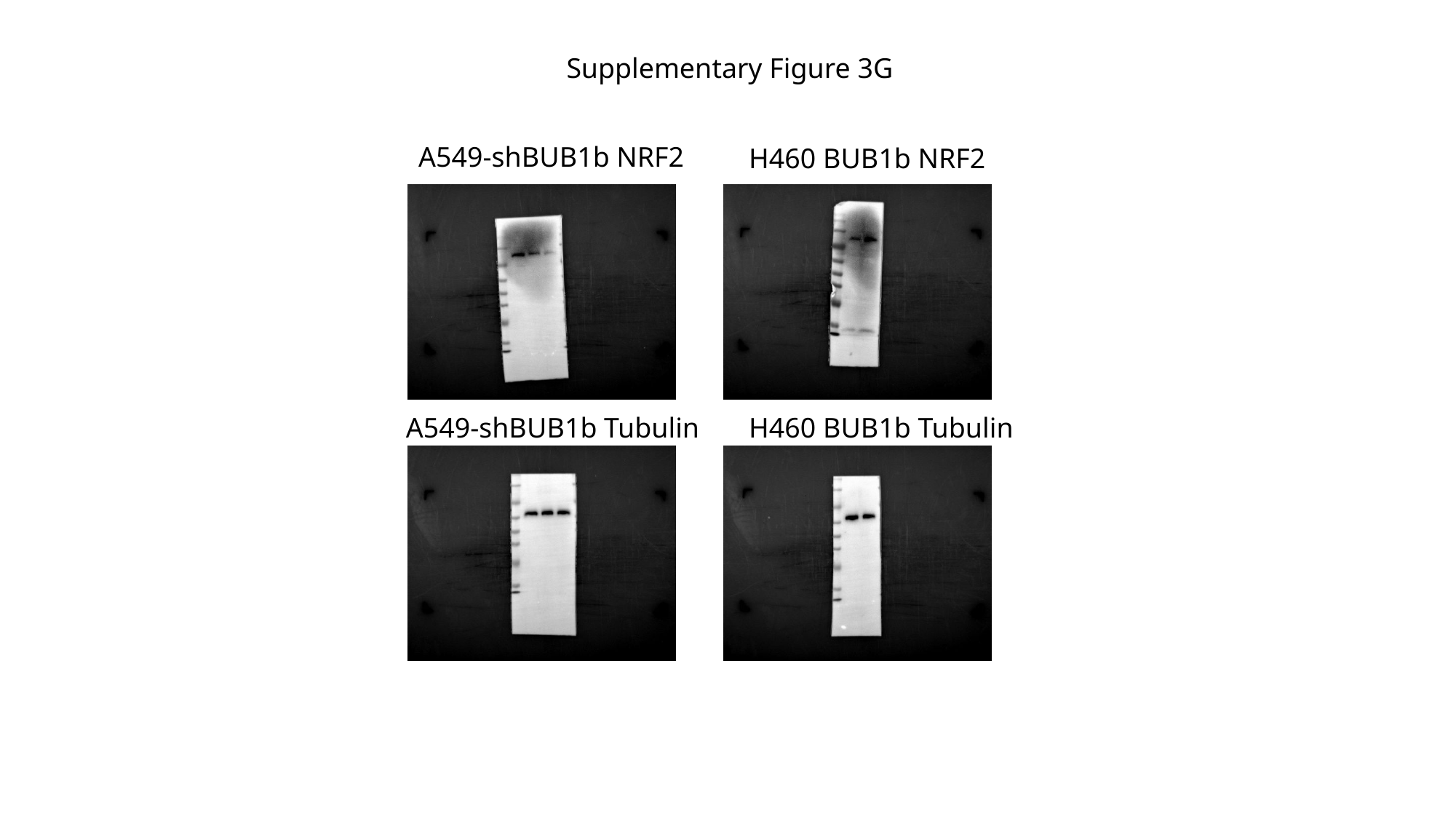

Supplementary Figure 3G
A549-shBUB1b NRF2
H460 BUB1b NRF2
A549-shBUB1b Tubulin
H460 BUB1b Tubulin

## Slide 11
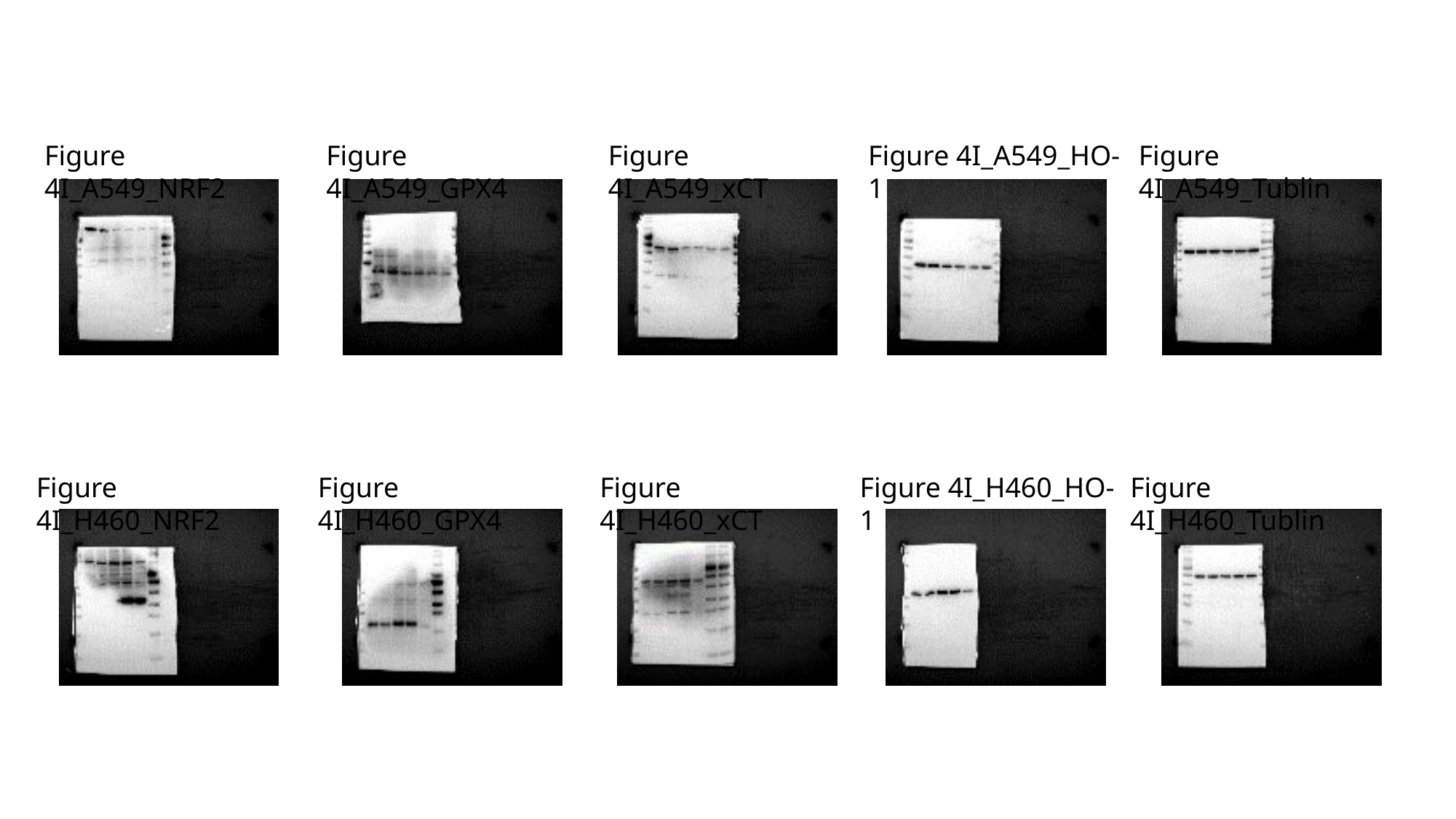

Figure 4I_A549_NRF2
Figure 4I_A549_GPX4
Figure 4I_A549_xCT
Figure 4I_A549_HO-1
Figure 4I_A549_Tublin
Figure 4I_H460_NRF2
Figure 4I_H460_GPX4
Figure 4I_H460_xCT
Figure 4I_H460_HO-1
Figure 4I_H460_Tublin

## Slide 12
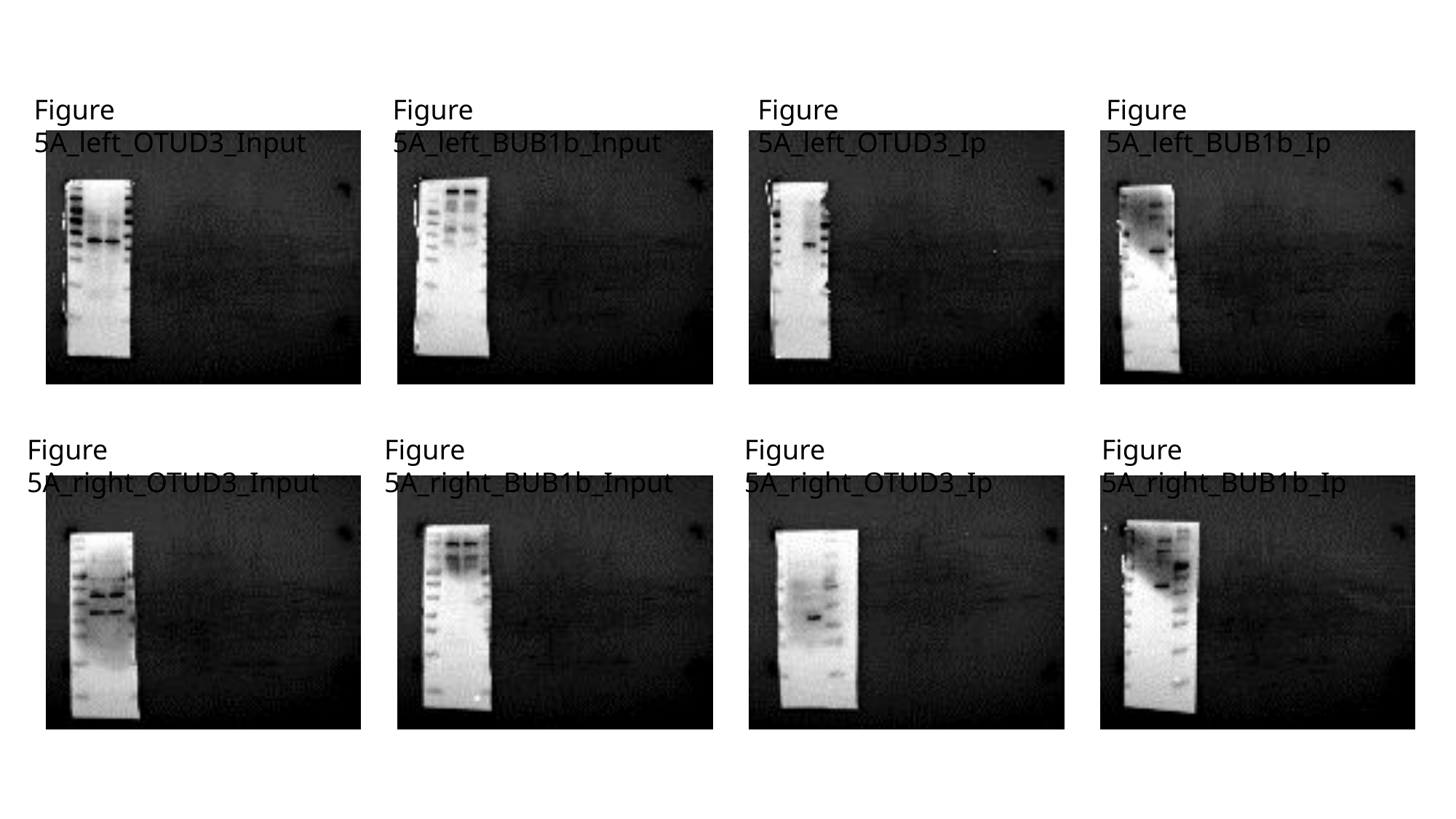

Figure 5A_left_OTUD3_Input
Figure 5A_left_BUB1b_Input
Figure 5A_left_OTUD3_Ip
Figure 5A_left_BUB1b_Ip
Figure 5A_right_OTUD3_Input
Figure 5A_right_BUB1b_Input
Figure 5A_right_OTUD3_Ip
Figure 5A_right_BUB1b_Ip

## Slide 13
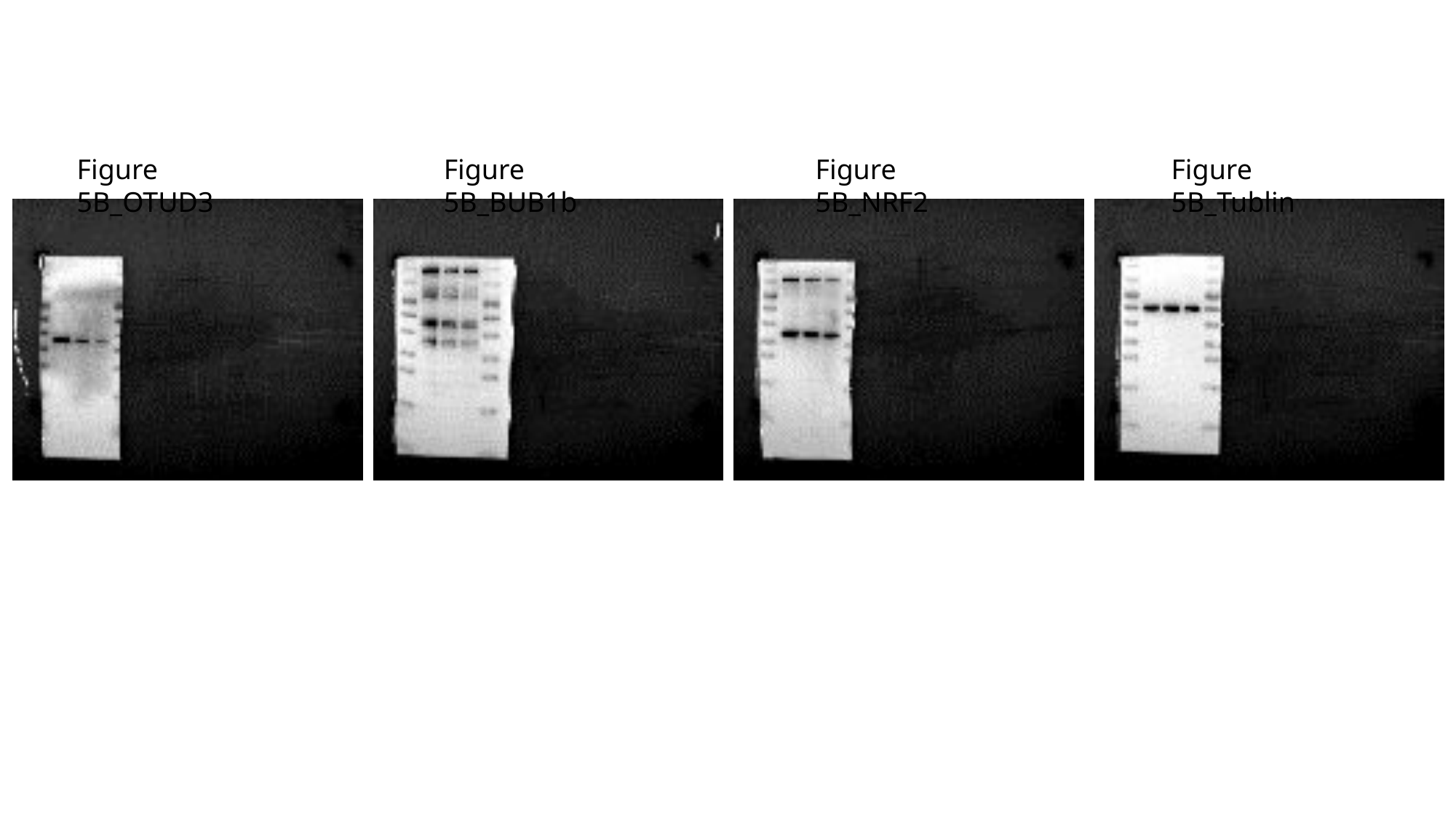

Figure 5B_OTUD3
Figure 5B_BUB1b
Figure 5B_NRF2
Figure 5B_Tublin

## Slide 14
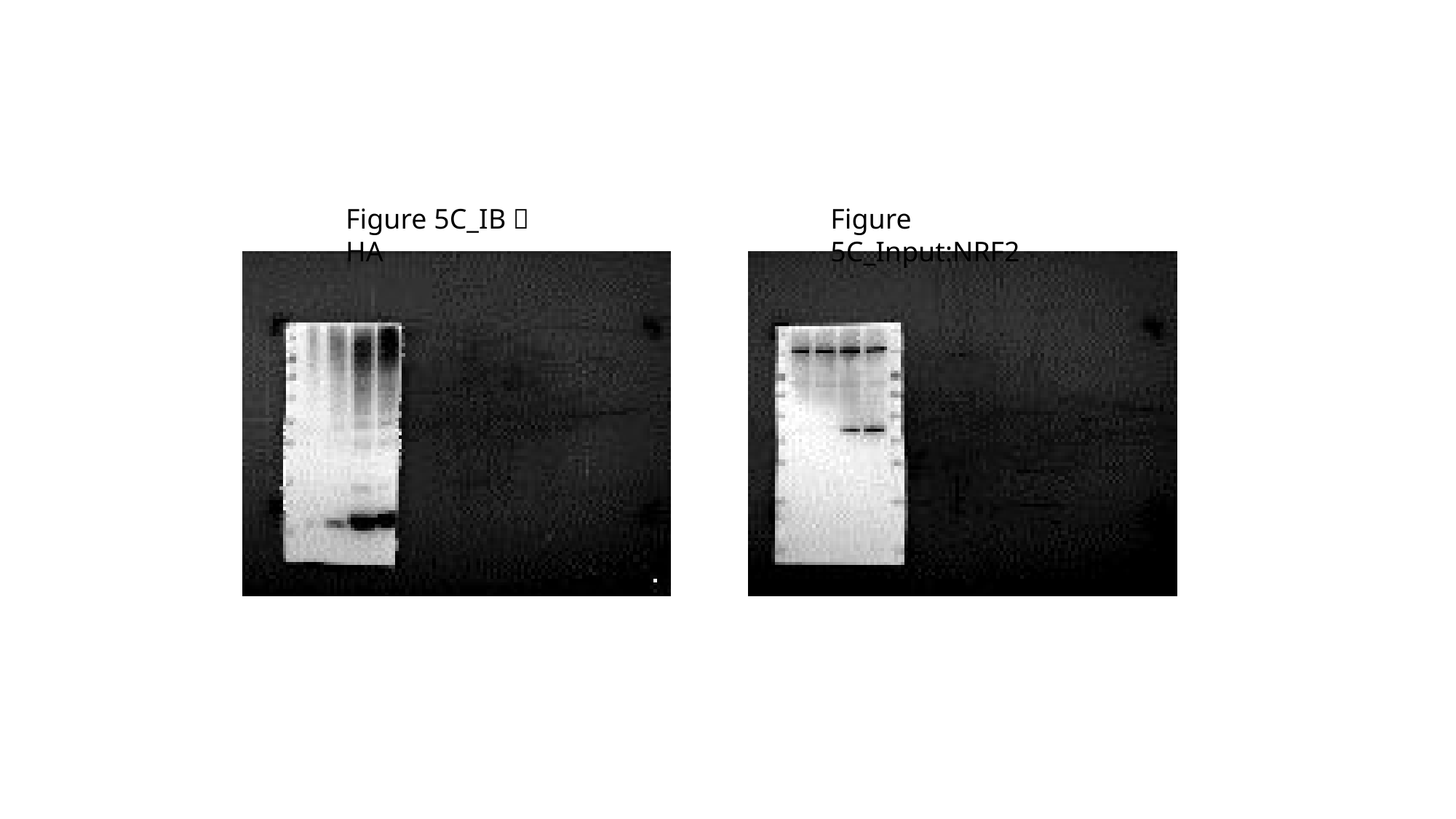

Figure 5C_IB：HA
Figure 5C_Input:NRF2

## Slide 15
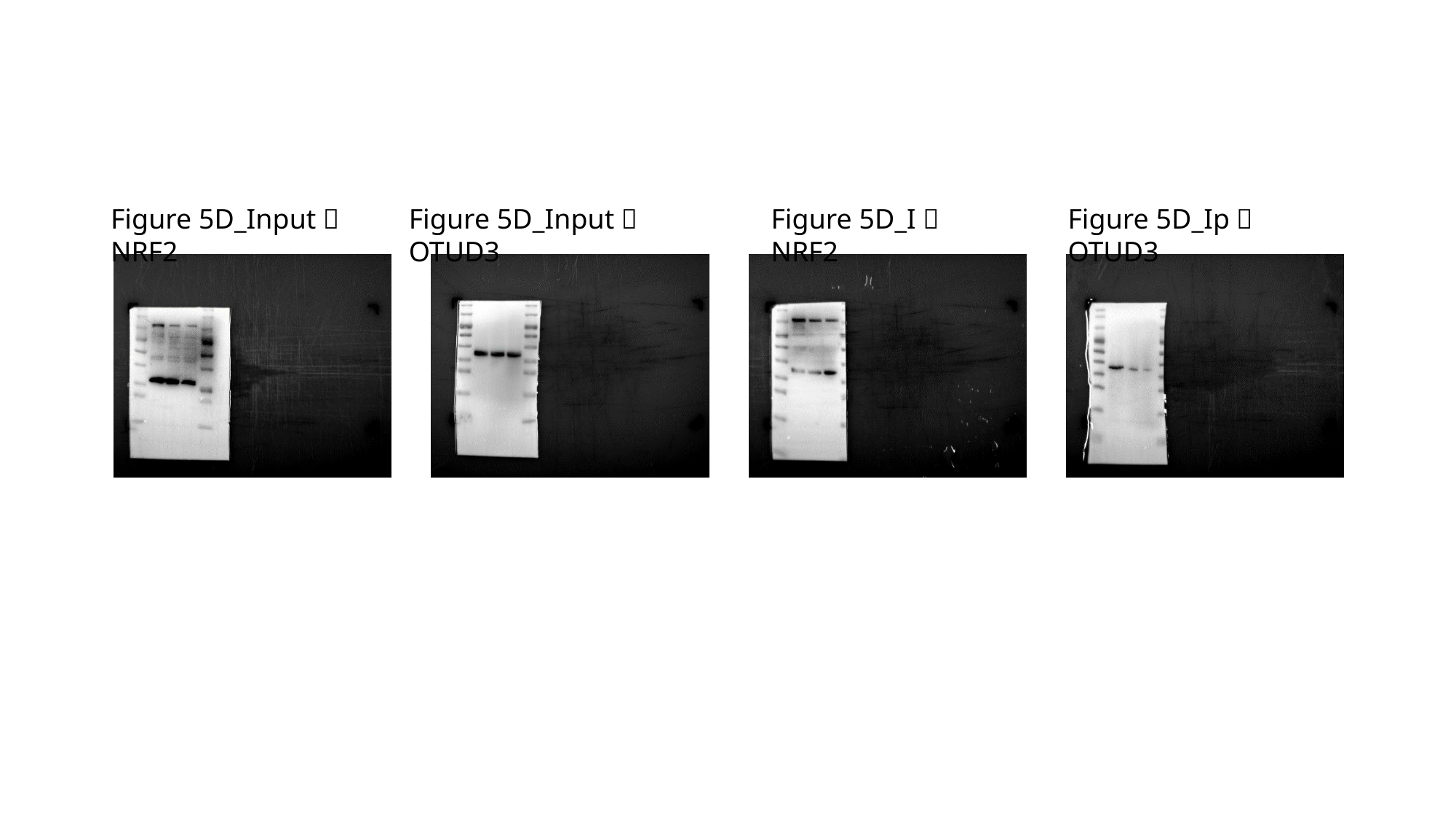

Figure 5D_Input：NRF2
Figure 5D_Input：OTUD3
Figure 5D_I：NRF2
Figure 5D_Ip：OTUD3

## Slide 16
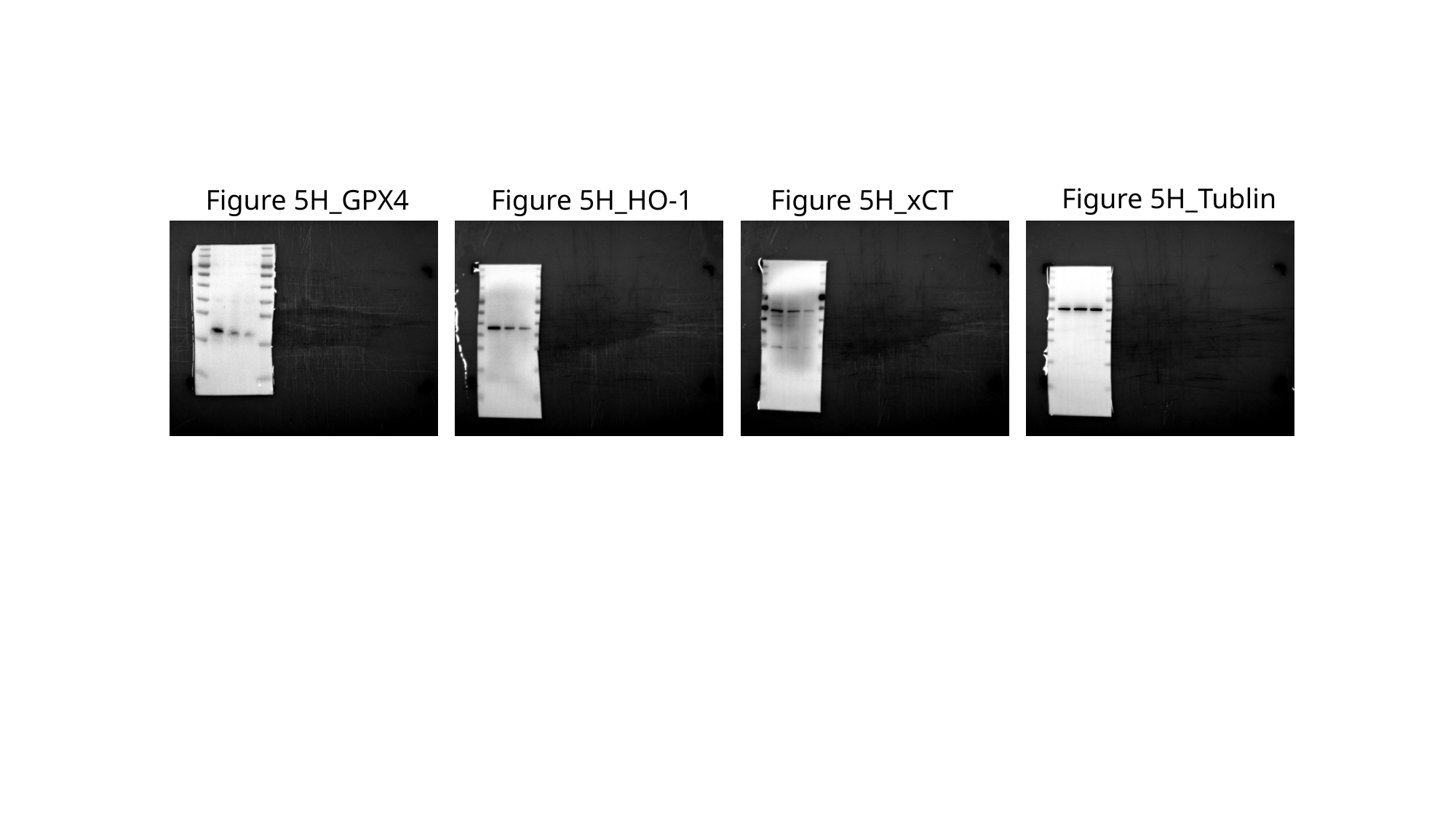

Figure 5H_Tublin
Figure 5H_GPX4
Figure 5H_HO-1
Figure 5H_xCT

## Slide 17
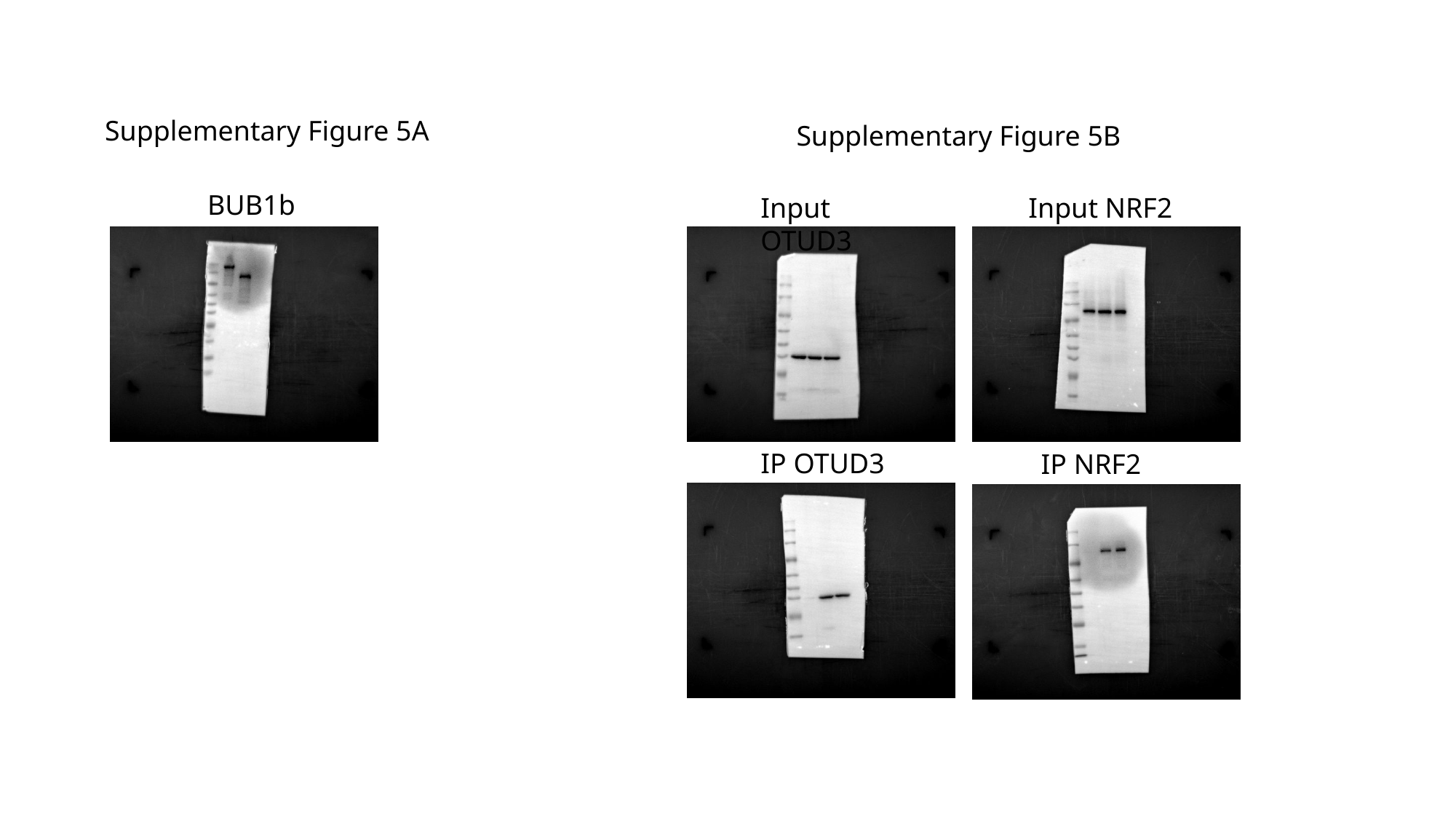

Supplementary Figure 5A
Supplementary Figure 5B
BUB1b
Input NRF2
Input OTUD3
IP OTUD3
IP NRF2
